# A Systems Approach to Predict Oncometabolites via Context-Specific Genome-Scale Metabolic Networks

**DOI:** 10.1371/journal.pcbi.1003837

**Published:** 2014-09-18

**Authors:** Hojung Nam, Miguel Campodonico, Aarash Bordbar, Daniel R. Hyduke, Sangwoo Kim, Daniel C. Zielinski, Bernhard O. Palsson

**Affiliations:** 1 Department of Bioengineering, University of California San Diego, La Jolla, California, United States of America; 2 School of Information & Communications, Gwangju Institute of Science and Technology (GIST), Buk-gu, Gwangju, Republic of Korea; 3 Centre for Biotechnology and Bioengineering, CeBiB, University of Chile, Santiago, Chile; 4 Severance Biomedical Science Institute, Yonsei University College of Medicine, Seodaemun-gu, Seoul, South Korea; 5 Department of Pediatrics, University of California San Diego, La Jolla, California, United States of America; Bar Ilan University, Israel

## Abstract

Altered metabolism in cancer cells has been viewed as a passive response required for a malignant transformation. However, this view has changed through the recently described metabolic oncogenic factors: mutated isocitrate dehydrogenases (IDH), succinate dehydrogenase (SDH), and fumarate hydratase (FH) that produce oncometabolites that competitively inhibit epigenetic regulation. In this study, we demonstrate *in silico* predictions of oncometabolites that have the potential to dysregulate epigenetic controls in nine types of cancer by incorporating massive scale genetic mutation information (collected from more than 1,700 cancer genomes), expression profiling data, and deploying Recon 2 to reconstruct context-specific genome-scale metabolic models. Our analysis predicted 15 compounds and 24 substructures of potential oncometabolites that could result from the loss-of-function and gain-of-function mutations of metabolic enzymes, respectively. These results suggest a substantial potential for discovering unidentified oncometabolites in various forms of cancers.

## Author Summary

Cancer and metabolism have been considered to be associated for a long period since Otto Warburg observed that tumor cells consume glucose and convert most of it to lactate, despite the presence of oxygen. However, the role of the Warburg effect in oncogenesis had been under doubt because no solid links between genetic variations in metabolic genes and cancer had been observed until recent days. When with the development of sequencing technologies, researchers found mutations in IDH1, IDH2 (isocitrate dehydrogenase) in medium-grade glioma and acute leukemia. As these mutated metabolic genes initiate unexpected enzymatic reactions, cancer cells show altered concentration of particular metabolites, here called “oncometabolites”. The oncometabolites regulate the epigenetic controls of cell differentiations. In this study, we predict potential oncometabolites that might originate from loss or gain-of-function mutations in nine types of cancer from massive scale cancer mutation data with a systems biology approach.

## Introduction

Otto Warburg observed that cancer cells convert most of their consumed glucose into lactate, despite the presence of sufficient oxygen [1]. This metabolic state, called “aerobic glycolysis” of cancer cells, has been viewed as a passive response required for a malignant transformation [2], and was originally hypothesized to be a necessary adaptation offsetting dysfunctional mitochondria. In contrast to this initial hypothesis, later studies have found that most tumor mitochondria are not defective in their ability to carry out oxidative phosphorylation [3]–[5].

**Figure 1:**
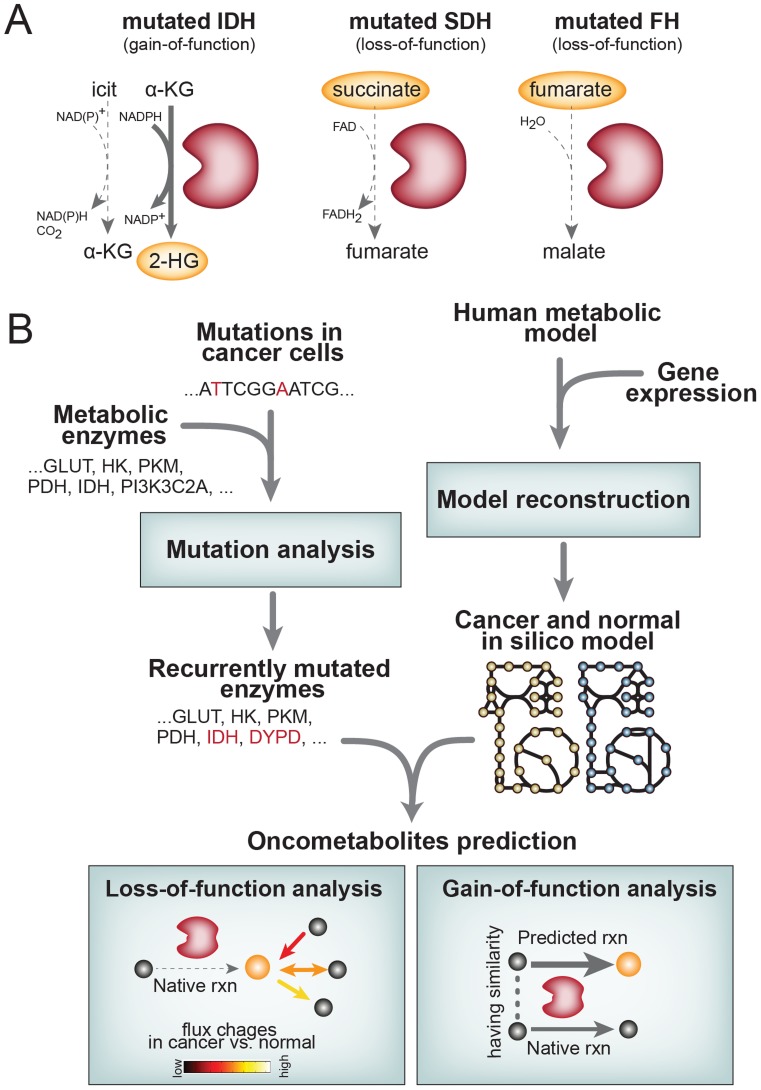
The origin of oncometabolites and an overview of the analysis workflow used. (**A**) (left) Mutant isocitrate dehydrogenases (IDH) enzymes show a neomorphic enzymatic activity to convert α-KG (α-ketoglutarate) into 2-HG (2-hydroxyglutarate), a small oncometabolite. The presence of mutant IDH1 or IDH2 proteins results in increased amounts of 2-HG, which then alters a number of downstream cellular activities. 2-HG competitively inhibits α-KG binding to several histone demethylases. (Middle, Right) Loss of succinate dehydrogenase (SDH) and fumarate hydratase (FH) enzymatic activity due to the mutation results in accumulated concentration of intracellular succinate and fumarate, respectively. (**B**) Overview of the work presented here. First, recurrently mutated enzymes which could produce potential oncometabolites are identified across the nine cancer types. Once the recurrently mutated enzymes were identified, oncometabolites were predicted by simulating flux changes between LoF cancer vs. normal *in silico* GEM (LoF analysis), and applying the chemoinformatics approach to predict promiscuous catalytic activities of enzymes resulting from their GoF mutations (GoF analysis).

The notion of passive cancer metabolism is being challenged by recent studies. It was shown that altered metabolism can by itself be a driver for oncogenic [6]–[10]. Recently characterized isocitrate dehydrogenase (IDH1, IDH2) mutations have established a new paradigm in oncogenesis in that the heterozygous point mutations confer a new metabolic enzymatic activity that produce an oncometabolite (e.g. 2-hydroxyglutarate (2-HG), from α-ketoglutarate) (**Figure 1A** **; left**). Surprisingly, 2-HG shows a 100-fold increased concentration in glioma and acute myeloid leukemia's (AML) patients with IDH1 or IDH2 missense mutations. This increased concentration of 2-HG competitively inhibits α-ketoglutarate (α-KG) binding to histone demethylases, thus blocking differentiation of cells [6], [7]. In parallel to IDH, loss-of-function mutations on succinate dehydrogenase (SDHA, SDHB, SDHC, and SDHD) and fumarate hydratase (FH) cause the accumulation of succinate and fumarate, respectively, which also acts a competitive inhibitor of α-KG-dependent oxygenases that regulate hypoxia-inducible factor (HIF) oncogenic pathway (**Figure 1A** **; middle, right**) [9]–[12]. Curiously, although IDH1 and IDH2 mutations are clearly powerful drivers of low grade glioma and AML, they seem to be rare or absent in other tumor types. This observation highlights the importance of the specific cellular context in understanding metabolic perturbations in cancer cells [6], [13].

Metabolism represents a complex network of biochemical reactions and it may be hard to decipher phenotypic consequences based on single reaction alterations. Annotated genomes and biochemical legacy data, however, have enabled the construction of genome-scale models (GEMs) of metabolism [14], [15] that have been successfully used to compute many observed metabolic states and properties [16]–[19]. A GEM is a formal and mathematical representation of reconstructed metabolism as a genome-scale network [18], [19], which consist of collections of metabolic reactions, their stoichiometry, the enzymes and the genes that encode them. GEMs offer a novel mechanistic link between genetic parameters and computed metabolic states. Two versions of the human metabolic reconstruction are available [14], [20].

With the rapid development of high-throughput experimental methods, recent integrative studies using disparate omics data types have deciphered characteristics of cancer metabolism using *in silico* GEMs [21]–[23]. Shlomi *et al*. dissected underlying principles of elevated glycolysis through the simulation of biomass production rates using GEMs [21]. Folger *et al*. identified drug targets for cancers based on synthetic lethal gene pair analysis using a generic GEM of cancer [22]. Further, the GEM analysis was applied to hereditary leiomyomatosis and renal cell cancer (HLRCC) to unravel the survival mechanism that enables the HLRCC cells to operate the mitochondrial electron transport chain despite mutations on FH [23]. The GEM of human metabolism has thus already shown its utility for the analysis and understanding of cancer metabolism.

In this study, we predict putative oncometabolites by incorporating genetic mutation information on a massive scale collected from more than 1,700 cancer genomes into context-specific GEMs of metabolism for nine cancer types. We reconstructed context-specific cancer and matching metabolic models from corresponding normal tissue for nine cancer types using gene expression profiles of primary cancer cells and site-matched normal cells. By integrating the exome mutation data source with the reconstructed GEM, we predict potential oncometabolites that could show altered concentration in cancer cells due to the loss- or gain-of-function mutations on enzymes (**Figure 1B**).

## Results

### Mutation information and gene expression data sets

We collected sets of exome mutation and gene expression data from The Cancer Genome Atlas (TCGA, http://cancergenome.nih.gov/), Cancer Cell Line Encyclopedia (CCLE) [24], and NCBI Gene Expression Omnibus (http://www.ncbi.nlm.nih.gov/geo/) (**Table 1**). We selected three cancer types (breast, kidney, and squamous cell carcinoma lung cancer) from the TCGA project with accompanying publically available genetic mutation and gene expression data sets. In addition, we collected mutation information for six types of cancers studied in the CCLE project (gastric, leukemia, liver, adenocarcinoma lung, ovarian, and pancreas). As the CCLE project does not provide gene expression for matched normal samples, we separately collected expression data of cancer and site-matching normal tissues from the NCBI GEO database by matching cancer histological types to the CCLE mutational data. Finally, nine unique histology types of cancer with mutation and gene expression data measured in cancer and site-matching normal were used in the present study (**Table 1**).

**Table 1:** Types of cancer and mutation and gene expression data sets used.

Cancer type	Mutation source	Expression source/ platform
(Histological sub type)	(No. samples)	(No. cancer/ No. normal), [reference]
Breast	TCGA	TCGA/ Illumina Hiseq RNAseqV2
(Invasive carcinoma)	(919)	(99/ 99)
Kidney	TCGA	TCGA/ Illumina Hiseq RNAseqV2
(Renal clear cell carcinoma)	(502)	(65/ 65)
Lung	TCGA	TCGA/ Illumina Hiseq RNAseqV2
(Squamous cell carcinoma)	(178)	(16/ 16)
Gastric	CCLE	GEO/ Affy HG U133 Plus 2.0 Array
(Adenocarcinoma)	(22)	(38/ 31), [GSE13911]
Leukemia	CCLE	GEO/ Affy HG U133A Array
(Acute myeloid leukemia)	(34)	(26/ 38), [GSE9476]
Liver	CCLE	GEO/ Affy HG U133A 2.0 Array
(Hepatocellular carcinoma)	(25)	(22/ 22), [GSE14520]
Lung	CCLE	GEO/ Affy HG U133A Array)
(Adenocarcinoma)	(45)	(28/ 28), [GSE7670]
Ovarian	CCLE	GEO/ Affy HG U133 Plus 2.0 Array
(Carcinoma)	(46)	(12/ 12), [GSE14407]
Pancreas	CCLE	GEO/ Affy HG U133 Plus 2.0 Array
(Carcinoma)	(44)	(36/ 16), [GSE16515]

### Large number of metabolic genes are mutated across different cancer types

**Figure 2:**
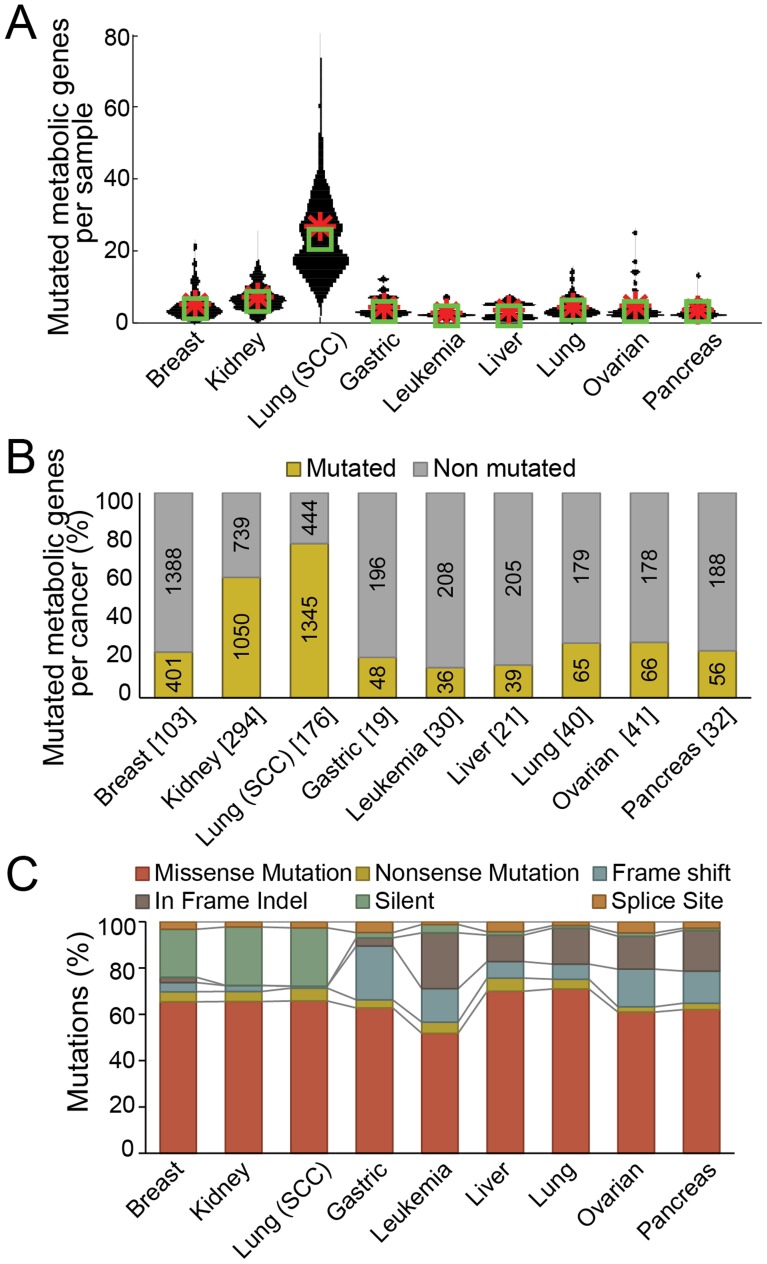
Overall statistics on genetic mutations in metabolic genes. (**A**) The violin plot depicts the distribution of the number of mutated enzymatic genes per patient (sample) in each cancer type (Green box: median, red asterisk: average). A median of 5∼20 metabolic genes were mutated per patient (sample). (**B**) The percentage of mutated metabolic genes per cancer. (**C**) The percentage of mutation types. Missense mutation was mostly frequently observed across cancer types.

To identify potential oncometabolites that originated from genomic variations in metabolic enzymes, we determined the total number of mutations in enzymatic genes across nine cancer types. The metabolic genes were identified from the global human metabolic network Recon 2 [14]. For every gene in Recon 2, the number of coding region genetic variants including classes of missense, nonsense, frame shift, in frame indel, silent, and splice site mutations were counted. Between 5 to 20 metabolic genes per sample were mutated in cancer cells (**Figure 2A**). Also, when we tallied the total mutation count per cancer type, the mutational frequency became more pronounced in each type of cancer (**Figure 2B**). In the CCLE data sets (gastric, leukemia, liver, ovarian, and pancreas), the initial mutation calling was made within a set of targeted metabolic genes. Thus we expect that the real number of mutated metabolic genes would be higher than the current numbers. Second, we confirmed that the missense mutation, which is the consensus type of mutation observed in IDH, was the most dominant mutation class in metabolic genes (**Figure 2C**). This analysis was also conducted on additional TCGA mutation data sets, which showed qualitatively similar results (**Table S1** in **Text S1**, **Figure S1** in **Text S1**).

### 20 enzymatic genes were selected for oncometabolite prediction

**Figure 3:**
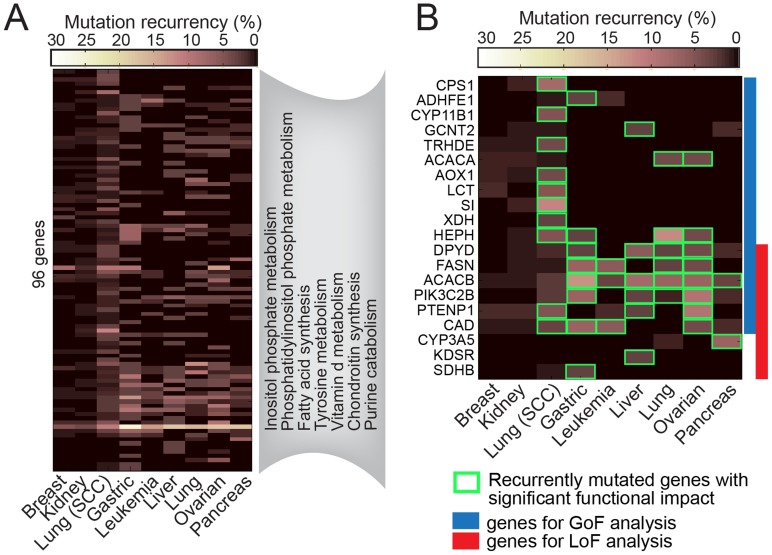
Recurrently mutated enzymes in the nine cancer types. (**A**) The 96 metabolic genes that are recurrently mutated in samples (≥ 5%). The functional pathways in the grey box denote enriched pathways among the 96 genes (Hypergeometric, *P* < 0.001). (**B**) The selected 20 metabolic genes that are recurrently mutated in samples (≥ 5%), and are expected to have a significant functional impact from their genomic sequence mutations in any of nine cancer types. Blue box: recurrently mutated genes for GoF analysis (gene having missense mutations). Red box: recurrently mutated genes for LoF analysis (genes having nonsense, frame shift indels, splice site indels&SNP mutations).

In order to predict oncometabolites that could originate from mutations, we selected metabolic genes that are recurrently found to be mutated in more than 5% of samples. Transporters were removed from our analysis, as they usually do not represent canonical metabolic transformations. As shown in the examples of IDH and FH, mutations could change the enzyme activity such that it gains a new function (gain-of-function) or loses its original function (loss-of-function). In this study, we divided the potential oncometabolites into two categories: (i) native oncometabolites that could change concentration due to the loss-of-function (LoF) mutations, and (ii) promiscuous oncometabolites that could change concentration due to the gain-of-function (GoF) mutations. Therefore, for this oncometabolites analysis, we classified mutations into two classes. First, we adopted a definition for LoF mutations to be correlated with loss of function: nonsense (stop codon introducing), splice site indels&SNP (splice site disrupting mutations), and frame shift indels (disrupting reading frame) [25] [26]. Second, we focused on the possibility of missense mutations as GoF mutations, which is the consensus type of mutation that is observed in IDH oncometabolite studies. Finally, the recurrently mutated genes are categorized into three classes: (i) recurrently mutated genes from a type of GoF mutation, (ii) recurrently mutated genes from types of LoF mutations (nonsense, frame shift indels, splite site indels&SNP) [26], and (iii) recurrently mutated genes from both of GoF and LoF mutations. As a result, we found 96 enzyme encoding metabolic genes that were recurrently mutated in the nine types of cancer (**Figure 3A** **, File S1**).

From the 96 recurrently mutated metabolic genes, we next selected genes that could have significant functional impacts on their catalytic activities due to the mutations. First, isoenzymes were filtered out because an unexpected mutational malfunction of one isoenzyme could be substituted by functions of other isoenzymes having duplicated catalytic activities. Second, mutated genes having smaller functional impact were removed. The impact of a mutation in the recurrently mutated genes was assessed by using the functional impact score (FIS) [27] which demonstrated a consistent higher accuracy in a recent systematic assessment [28]. A FIS is derived from multiple sequence alignments of amino acid sequence homologs, thus, the score is based on the evolutionary conservation of a mutated residue in a protein family. By applying the aforementioned two criteria, 20 metabolic genes were selected for detailed analysis. These 20 genes are recurrently mutated in samples (≥ 5%), and are expected to have significant functional impact from their genomic sequence mutations (**Figure 3B** **, Figure S2** in **Text S1, File S1**). Notably, we observed mutation recurrence in LoF SDHB that is one of the previously identified oncometabolite-producing enzymatic genes (**Table S2**, **S3** in **Text S1**).

### Reconstructed cancer and normal metabolic GEMs correctly characterize *in vivo* cellular metabolic activity

In order to predict oncometabolites originating from LoF mutations on enzymes, we simulated flux changes of reactions in a cancer cell using GEMs. For the reconstruction of cancer specific and matched normal metabolic networks, gene expression data sets of cancer and matched normal were used. Here, the specific characteristics of cancer and normal metabolic models were represented by the rerouted network structure based upon the presence or absence of an enzymatic reaction in the intracellular environment of cancer and normal cells. This presence or absence of reactions was determined by the present/absent (P/A) calls of enzymatic genes (see **Materials and Methods** for details).

With the result of P/A calls of enzyme-encoding genes in nine cancer and normal gene expression data sets, we evaluated the relevance of P/A call results to the cancer specific metabolism. Here, we first assessed gene-wise P/A alterations in cancer vs. normal across nine cancer types. In this analysis, we confirmed that the P/A alterations in cancer vs. normal of enzymatic genes were not consistent across different cancer types (**Figure S3A** in **Text S1**). This inconsistency is in accordance with previous finding that the expression differences of individual genes vary from cancer to cancer [29]. However, when the alterations were evaluated at the level of functional pathways, several pathways related to common malignancy features showed significant P/A alterations (**Figure S3B** in **Text S1**). A pathway is considered to be significantly altered if the pathway is found to have a significantly higher number of genes with P/A alterations than the corresponding number found in a random set of genes (see **Text S1** for details). Specifically, reactions transporting (exporting, secreting) substances inside and outside of the cell were frequently altered across nine types of cancer. Furthermore, we confirmed that these altered pathways had significant over representation of mutations (**Figures S3C-S3E** in **Text S1**). We reconstructed nine cancer and normal matching models using P/A gene expression calls the Gene Inactivity Moderated by Metabolism and Expression (GIMME) algorithm (see **Materials and Methods** for details, **Table 2** **, Figures S4-S6** and **Tables S4-S6** in **Text S1**).

**Table 2:** Summary of the reconstructed cancer and normal GEMs.

Cancer type (Histological sub type)	Cancer				Normal			
	# TR	# GAR	# TG	# TM	# TR	# GAR	# TG	# TM
Breast (Invasive carcinoma)	6602	3408	1069	4782	6712	3518	1166	4820
Kidney (Renal clear cell carcinoma)	6730	3536	1118	4865	6834	3640	1212	4899
Lung (Squamous cell carcinoma)	6753	3559	1168	4818	6878	3684	1275	4865
Gastric (Adenocarcinoma)	5938	2750	806	4626	5643	2455	805	4690
Leukemia (Acute myeloid leukemia)	5119	1931	503	4242	5322	2134	671	4325
Liver (Hepatocellular carcinoma)	5926	2738	869	4725	5972	2784	832	4755
Lung (Adenocarcinoma)	4737	1549	480	4235	5494	2306	695	4480
Ovarian (Carcinoma)	4590	1402	494	4172	5177	1989	779	4465
Pancreas (Carcinoma)	6439	3251	991	4810	5860	2672	883	4609

# TR: number of total reactions; # GAR: number of gene associated reactions; # TG: number of total unique genes; # TM: number of total metabolites.

The accuracy of the cancer and normal matched metabolic models were evaluated based on: (i) how well the structure of the reconstructed network represents gene expression data, and (ii) how well the simulated fluxes predict metabolic states of cancer and normal cells.

**Figure 4:**
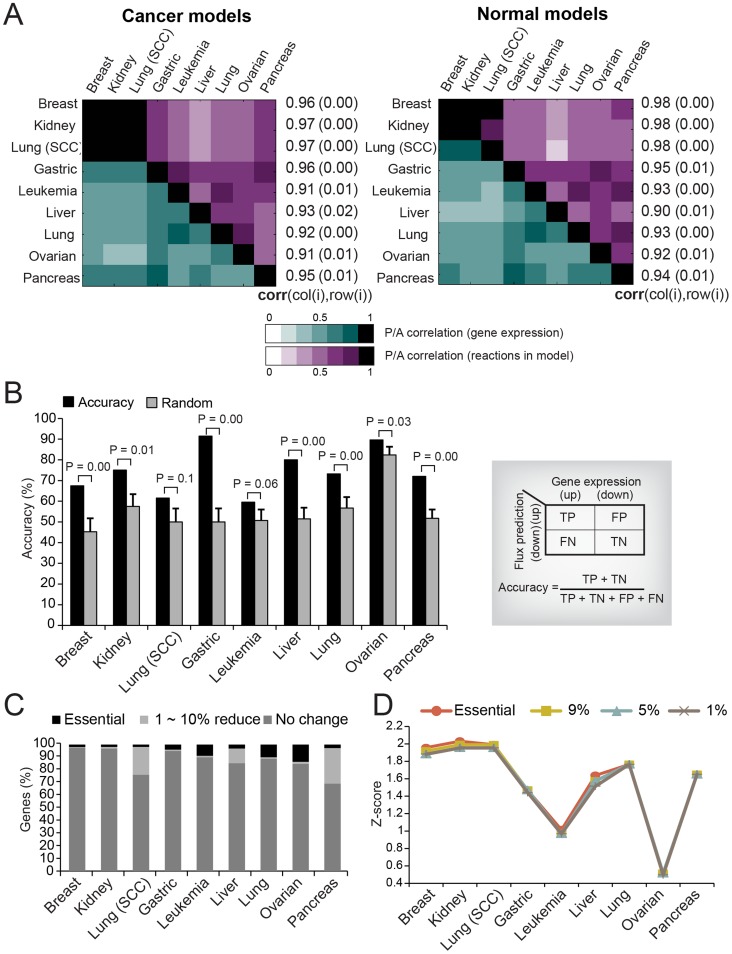
The accuracy of the reconstructed context-specific GEMs. (**A**) Pearson's pairwise correlation plot shows correlation of gene expression P/A calls (right-bottom; green) and model structures (left-upper; purple) between cancer types. Values next to the correlation plot represent final correlation between gene expression P/A calls and the network structure for each cancer type, values in parentheses represent P-values of significance (**Figure S6** in **Text S1**). (**B**) The accuracy of the predicted flux changes from the reconstructed models. The prediction of gene expression changes from changes in flux was tested against gene expression data sets for cancer vs. normal. The black bar depicts the accuracy of the flux prediction from the reconstructed models. The grey bar denotes the median value of 10,000 accuracies from the random flux. The error bar shows the standard error mean (SEM) of 10,000 random accuracies. (**C**) The percent stacked column chart shows the percent of genes for three categories of gene knockout effects (Essential: knockout effect that reduced the growth rate more than 10%, 1∼10% reduced: knockout effect that reduced the growth rate from 1 to 10%, No change: knockout does not cause any change). (**D**) The accuracy of a model is evaluated by enrichment analysis of experimentally validated essential genes among the predicted essential genes with the model. A P-value from the hypergeometric test is converted to a z-score. Higher z-scores correspond to more significant enrichment (Orange: z-scores of essential genes, yellow: z-scores of genes causing 9% growth reduction, sky-blue: causing 5% growth reduction, 1%: causing 1% growth reduction).

Since the characteristics of the reconstructed model are mainly determined by the result of gene expression P/A calls, we first evaluated how well the network structure of the model represents the gene expression P/A calls. Since a gene can be associated with multiple reactions, or vice versa, the correlation between P/A calls and the network structure was calculated in two steps. The final correlation was determined by correlations of two vectors of Pearson's correlation coefficient (PCC) calculated from pairwise correlations of presence/absence of gene expression and pairwise correlations of presence/absence of reactions between cancer types (**Figure 4A**). Correlation coefficients varied from 0.90 to 0.98, and the PCC values were significantly higher than random PCC values (**Figure S7** in **Text S1**). Thus, the reaction content of the cancer and normal models represent the gene expression data sets well.

Second, we tested the accuracy of the simulated flux states of the reconstructed models by evaluating whether the flux states correctly predict metabolic states of cancer and normal cells. We hypothesized that if the flux through a reaction was predicted to increase (or decrease) its magnitude in cancer compared to normal, then the expression level and/or abundance of the associated active enzyme in cancer will increase (or decrease) in order to meet the change in flux. With this hypothesis, we compared the changing pattern (increase or decrease) between flux and gene expression for the reactions with a significant flux change. Flux changes were calculated using the Markov chain Monte Carlo (MCMC) (see **Materials and Methods** for details). **Figure 4B** shows the accuracy of the predictions for the significantly changed fluxes (P-value < 0.001, fold-change ≥ 2). The results are fairly accurate. Among the nine cancer models, seven cancer types, except the lung (SCC) cancer and leukemia, showed significantly better accuracy than random tests (P-value < 0.05). Furthermore, our results were qualitatively robust with variations in the P-value and fold-change thresholds (**Figure S8** in **Text S1**)

In addition, we evaluated the accuracy of predicting essential genes. The accuracy of reconstructed models was evaluated by the enrichment of *in vivo* essential genes among *in silico* essential genes computed from Flux Balance Analysis (FBA) (see **Materials and Methods** for details). Across the nine cancer types, we found that about 3–30% of genes contributed to cell growth, about 2–14% of genes were essential for the cell growth (**Figure 4C**). Further, in several cancer types (breast, kidney, lung (SCC), and liver), we found that genes predicted to have higher contributions to the biomass formation are more enriched in the *in vivo* essential genes (**Figure 4D**).

The analyses presented here confirm that the simulated flux through the reconstructed models effectively predicts *in vivo* cellular metabolic activity of cancer and normal cells.

### 15 candidate oncometabolites are predicted as a result of loss-of-function (LoF) mutations

From the substrates and products of the nine LoF mutant enzymes (**Figure 3B**), we chose the metabolites that significantly change the flux state in cancer cells with LoF mutation activity relative to the corresponding normal tissue. These metabolites were identified as potential oncometabolites associated with LoF mutations.

To predict LoF oncometabolites, we first modified a reconstructed cancer model into mutated enzyme deficient models. For example, as shown in Figure 3B, FASN, ACACB, and CAD genes were found to be recurrently mutated in leukemia. For each gene, we built a corresponding leukemia model with a deficiency in that gene (e.g.. FANS-deficient, ACACB-deficient, and CAD-deficient leukemia models). We built a total of 13 mutated enzyme deficient models for nine genes across nine cancer types that have feasible flux solution states with the deficient function of the mutated enzyme (**Figure S9** in **Text S1**).

**Figure 5:**
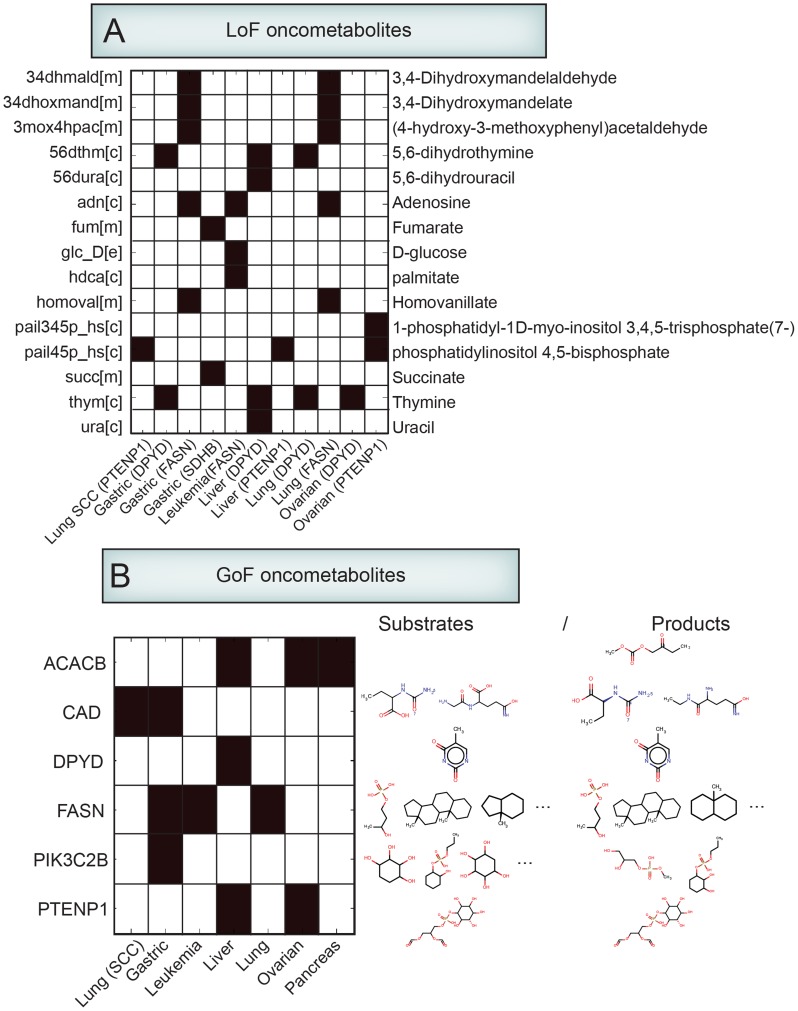
Predicted oncometabolites. (**A**) LoF oncometabolites. Metabolites surrounded by reactions with significantly changed flux in the mutated enzyme deficient model compared to the normal model were finally predicted as the potential LoF oncometabolites. The heatmap shows predicted oncometabolites (black) across nine mutated enzyme deficient cancer models ([m], [c] and [e]: metabolites observed in mitochondria, cytosol, and extracellular, respectively). (**B**) GoF oncometabolite substructures. By applying the chemoinformatics approach, promiscuous catalytic activities of enzymes resulting from their GoF mutations were predicted. The heatmap represents predicted dominant substructrues of promiscuous substrates and products across seven cancer types. Full list of substructrues of promiscuous substrates and products is shown in the Table S8 in Text S1.

Once the deficient models were built, we simulated flux changes that result from the enzyme deficiency by employing an MCMC sampling method to the enzyme deficient models and matching normal (no cancer with non-enzyme deficient) models. Substrates or products of reactions with significantly changed flux for deficient models as compared to normal models were chosen as potential oncometabolites. Finally, 15 unique metabolites catalyzed by the mutated enzymes and surrounded by significantly changing flux were predicted as context-specific LoF oncometabolites (**Figure 5A**). Notably, previously known oncometabolites, succinate and fumarate, were predicted as potential oncometabolites due to the LoF of SDBH in gastric cancer (**Figure 5A**, **Table S7** in **Text S1**) [9]–[12].

### 24 dominant substructures of candidate oncometabolites are predicted as a result of gain-of-function (GoF) mutations

As described in the example of IDH, mutated enzymes could possibly confer new catalytic activities by simultaneously changing the native reaction mechanism and/or catalyzing different substrates. In this study, we used a chemoinformatics approach to predict promiscuous catalytic activities of enzymes resulting from GoF mutations. This chemoinformatics approach has been shown to be useful in predicting enzymes promiscuity [30], [31], assigning Enzyme Commission (EC) number [32], [33], and analyzing reaction databases [34].

To predict candidate oncometabolites resulting from promiscuous activities of mutated enzymes, a systems framework was developed to determine new enzyme functionalities due to mutated enzymes. First, each substrate and product present in the mutated enzyme reactions were compare against the Human Metabolome Database (HMDB) [35]. In order to decrease computational efforts and select a manageable set of metabolites for biochemical reaction operators (BROs) simulation, Tanimoto coefficient cut off values were determined for each mutated enzyme reactions. Then, synthetic reactions were constructed by applying generic BROs to previously HMDB selected metabolites (see **Materials and Methods** for details). Then, simulated synthetic reactions were compared against the reactions associated with the mutated enzymatic genes. To do this, Tanimoto coefficients of substrates and reactions were calculated between pairs of reactions. In order to emulate the GoF enzyme behavior, different Tanimoto coefficient cut-off values were imposed on each reaction according to the IDH GoF mutation case. Pairs of reactions with Tanimoto similarity scores of less than or equal to a specific cut-off were saved and identified as possible gain of function pairs (see **Figure S10** in **Text S1**for details). Finally, from these reaction pairs, structural features of oncometabolites were predicted.

Among the 17 genes with recurrent GoF mutations, seven genes could have catalytic activity in cancer (see **Figure S11** in **Text S1** for details). The final GoF mutated enzymatic gene list for this analysis contains seven unique genes. Among the reactions associated with the seven mutants, reactions catalyzing large molecules that could yield more than 30,000 promiscuous activities due to the compounds complexity were excluded from the analysis, and finally 33 reaction associations were identified from the enzymatic list. Notably, cofactors were not taken into account for the compound list generation.

A summary of mutated enzymes associated reactions and their predicted promiscuity catalytic activities is given in Table S8 in Text S1. For example, for the CAD gene which catalyzing L-glutamine, 2866 potential promiscuity catalytic reactions associated with 170 substrates and 1644 products were predicted in CAD mutant lung (SCC) cancer and gastric cancer (**Table S8** in **Text S1**, **Figure 5B**, all detailed information of predicted promiscuity reactions is shown in the **File S2**).

In most cases promiscuous substrates show similarities with the native substrate [36], [37]. We conducted compound similarity analysis in order to demonstrate dominant substructures of promiscuous substrates and products (**Table S8** in **Text S1**). The compound structure shown in the Table S8 in Text S1 is the dominantly observed substructure of promiscuous substrates and products. Notable, several promiscuous substrates and products did not have dominant substrates. Finally, we identified 24 promiscuous compound substructures as features of GoF oncometabolites (**Figure 5B**).

## Discussion

In this study, we predicted potential oncometabolites in nine types of cancer by analyzing the massive scale genetic variants integrated with cancer and normal GEMs. We first predicted potential oncometabolites that could result from LoF mutations by simulating flux changes in the metabolic network due to the LoF of mutated enzymes. Second, we predicted oncometabolites that could result from GoF mutations by inferring promiscuous catalytic activities of enzymes resulting from their GoF mutations. Setting aside the generally accepted LoF criterion (nonsense, splice-variants and frame-shift), we rarely have information about the mutational directionality of a given missense mutation whether it functions as GoF or LoF. Specifically in the oncometabolite analysis, enzymes can be disrupted in both ways. Whereas an LoF of an enzyme gives a predictable malfunctions (e.g. an accumulation of a target product), a GoF mutation is much more unpredictable and is the very place in which an *in silico* analysis should be targeted. As in the example of IDH, IDH gained an unexpected catalytic activity that is result from missense mutations. Therefore, in this study, we focused on the possible metabolic perturbation of a GoF mutation in an enzyme can raise. Note that, assuming a mutation to be GoF is different from asserting the mutation is GoF. Using the aforementioned criteria, we predicted 15 oncometabolites resulting from the LoF mutations, and 24 substructures of oncometabolites resulting from the GoF mutations. These predictions can be used as a guide to examine select mutant enzymes and generation of oncometabolites. Notably, in our reconstructed models, several cancer and/or normal models do not uptake glucose or secrete CO_2_ in their optimal flux states (optimal solution). In order to prevent this problem, we allowed small amounts of uptakes to important vitamins exchange reactions (**Table S4** in **Text S1**). Also, we constrained models to uptake glucose, oxygen and secrete CO_2_, biomass. With the updates, now most of models produce presumably reasonable uptake and secrete flux states (see flux variation ranges in the **Table S5, S6** in **Text S1**). Although still several models do not secrete any CO_2_ in the optimal flux states, this problem is not a critical problem in our study since the representative flux states in our results were determined from the sampling points within the suboptimal solution space (90% of optimality solution space) that is the flux variation ranges shown in the Table S5 and S6 in Text S1.

Altered energy metabolism in cancer cells is accepted as a hallmark of cancer [2], [5]. With the discovery of oncometabolites such as 2-HG, succinate, and fumarate that originate from mutations in key enzymes, alteration of metabolism is now considered to be a strong oncogenic factor. With the existence of oncometabolites established, there is clearly a great interest in determining if there are additional metabolites with oncogenic potential. Large-scale data sets available for a variety of cancers and genome-scale models of metabolism can be used to predict the existence of oncometabolites, and can contribute to therapies and biomarkers in cancer.

Oncometabolites could be used to develop therapies and identify biomarkers associated with cancer. Recent studies showed that inhibition of mutant IDH1 delays growth of glioma cells and induces cellular differentiation in leukemia, which shows possibility of a potential application as a therapy for cancer [38], [39]. Also, it has been reported that a breast cancer subtype with elevated level of 2-HG was associated with reduced survival. This study indicated that high levels of 2-HG may be a useful biomarker for breast cancer diagnosis and prognosis [40]. Thus, predicting existence of potential oncometabolites would be beneficial in cancer therapies and biomarker identification.

## Materials and Methods

### Model setup and parameterization

In order to reconstruct cancer type specific metabolic models and their paired normal models, we used data sets of gene expression experiments collected from TCGA consortium and GEO Database which are composed of expression profiles of primary cancer cells and site-matched normal cells (**Table 1**). In order to maximize platform consistency, we focused on using Affymetrix and Illumina Hiseq RNA-seq platforms. The present/absent (P/A) calls of the Affymetrix gene expression were made using the ‘mas5calls’ function in the ‘affy’ package (ver. 1.28.0) implemented in R (ver. 2.15.0), and a threshold of 10 read maps was used to define detection of the P/A calls of RNA-seq data at the gene level [41]. In our study, genes that are expressed in more than 99% of the total number of samples in a data set are finally determined as present (expressed) genes.

The P/A call results of gene expression are then incorporated to the reactional space using Gene Inactivity Moderated by Metabolism and Expression (GIMME) algorithm implemented in COBRA Toolbox v2.0 [42], [43]. For the medium condition, a standard RPMI-1640 condition was used in all simulations [21], [22]. Although, same medium uptake rates were applied to different cancer and normal models, this is not likely to cause serious artifacts since the proposed method is based on differences of network structure of cancer vs. normal models as determined by gene expression P/A call results.

As cancer cells are known to maximize their proliferation rate, the biomass formation was maximized in all cancer models [21], [22] (**Formula 1**). However, normal cells may have different objectives depending on the growth signals [3], thus we designed a new objective function which can cover the different cellular objectives in both proliferative and quiescent normal cells (**Formula 2**).
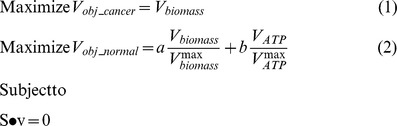



Because normal cells have different, or multiple, physiological objectives (i.e., biomass or ATP formation), we constructed an objective function that was a linear combination of these physiological functions (**Formula 2**). Each reaction flux in the objective function was scaled by its maximum achievable flux in the given growth condition. The contribution of each component may be weighted with an additional coefficient (*a* and *b*) with a value of 0 denoting no contribution to the objective. Here, coefficient *a* and *b* are set to be 1 and 1, respectively (equal contribution of biomass and ATP).

### Markov chain Monte Carlo sampling

The distribution of feasible fluxes in the models used in this study was determined using Markov chain Monte Carlo (MCMC) sampling [44], and was implemented with the COBRA Toolbox v2.0 [42]. Specifically, the objective function was provided a lower bound of 90% of the optimal growth rate as computed by flux balance analysis [45]. Thus, the sampled flux distributions represented sub-optimal flux-distributions, but still simulated fluxes relevant to cell growth and maintenance.

MCMC sampling was used to obtain thousands of feasible flux distributions using the artificially centered hit-and-run algorithm with slight modifications, as described previously [46], [47]. As a result, a mixed fraction of approximately 0.50 was obtained, suggesting that the space of all possible flux distributions is nearly uniformly sampled.

For each reaction, a distribution of feasible steady-state flux values was acquired from the uniformly sampled points, subject to the network topology and model constraints. Similar measures were taken for all other models in this work.

### Flux change prediction in cancer versus normal

To simulate changes in reaction flux occurring in a shift between cancer and normal, the sampled fluxes for each reaction were compared between cancer and normal as follows. First, reactions that carried no flux in both conditions or that were involved in loops [48] were removed and not used in further analysis. Next, flux magnitudes were normalized between each pair of media conditions. To do this, the flux value of each sample point was divided by the sum of all flux magnitudes for the sample point (**Formula 3**).
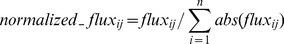
(3)

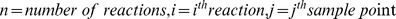



Once the flux values were normalized, the changes of fluxes between two conditions were determined as previously described [47]. Briefly, differential reaction activity was determined by assuming that a reaction is differentially activated if the distributions of feasible flux states (obtained from MCMC sampling) under two different conditions do not significantly overlap. For each metabolic reaction, a P-value was obtained by computing the probability of finding a flux value for a reaction in one condition that is equal to or more extreme than a given flux value in the second condition. The significance of P-values was adjusted for multiple hypotheses (FDR ≤ 0.01).

### Flux versus gene expression changes

The approach presented here extends the pre-published method called Flux Space Shift analysis (FSS) [49]. FSS utilizes MCMC sampling of the metabolic solution space to compute the distribution of all possible steady-state fluxes an enzymatic reaction can carry in a cell in a given growth condition. For each reaction, a P-value is computed from the distributions of possible fluxes for the reaction in a cancer cell and its matched normal cell. This P-value represents the probability of choosing a flux value from a reaction in a cancer cell that is also within the distribution for that reaction in a normal cell. The P-values are then corrected for multiple hypotheses, and the list of reactions that show significantly different fluxes for cancer vs. normal is returned, along with the direction of the change in magnitude (up or down). All significantly changed fluxes are then decomposed into a list of genes that help to catalyze the reactions using the gene-protein-reaction associations in the model. Through this, one can obtain lists of genes that are expected to be up-regulated or down-regulated. Here, in order to minimize the ambiguity, genes that are associated both with reactions that increase and other reactions that decrease were removed, and reactions catalyzed by more than one isoenzyme were also filtered out from the analysis since not all isozymes are necessarily to change their gene expression level. Once the lists of genes that are expected to be up- or down-regulated are gathered, the predictions were compared to the actual gene expression changes. The accuracy of the prediction was demonstrated by the ratio of the number of correctly predicted genes divided by the number of total predictions (**Formula 4**). The significance of the accuracy is demonstrated by a comparison with the background distribution using 10,000 random accuracy tests. 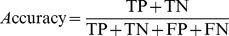
(4)(TP: True positive, TN: True negative, FP: False positive, FN: False negative)

### Gene essentiality prediction

Accuracy of reconstructed cancer specific models was assessed using experimentally determined essential genes. We collected a list of cancer essential genes from [50]. Ultimately, 14 cancer cell essential metabolic genes which were reported as common essential genes across 13 cancer cell line were used.

We performed *in silico* single gene deletion tests on each model, and genes whose deletion effect reduces the maximum objective reaction by more than 10% were determined to be *in silico* essential genes. Finally, as the total number of predicted *in silico* essential genes varies according to the models, the accuracy of the models were evaluated with P-values from the hypergeometric enrichment tests of experimental essential genes against the *in silico* essential genes.

### Gain-of-function oncometabolite prediction

#### Defining similarity (measuring similarities of substrates and reactions)

Mutated enzymes are able to carry out new catalytic functions by simultaneously changing the native reaction mechanism and catalyzing different substrates. The changes could be measured and compared using a chemoinformatic approach. Previous studies have successfully shown the efficacy of the chemoinformatic approach for enzyme promiscuity predictions [30], [31], reaction Enzyme Commission (EC) number assignments [32], [33], and reaction database analysis [34]. In this study, native and nonnative substrates and reactions are represented by a fingerprint, and the similarity of reactions and substrates were compared by calculating the corresponding Tanimoto coefficient between fingerprints.

#### Chemical representation

For compound and reaction representation we used MDL Molfiles. A Molfile contains information about the atoms, bonds, connectivities, and coordinates of a molecule. The Molfile consists of some header information, the Connection Table containing atom info, then bond connections and types, followed by sections for more complex information.

#### Substrate fingerprint

As stated before, substrates are represented by chemical fingerprints. The chemical fingerprint (CFP) of a molecule is defined as CFP  =  (F*i*), in which F*i* refers to a molecular fragment with real occurrences of a molecule. F*i* is obtained by molecular fragmentation method. Each F*i* in the fingerprint is represented in bit string where each position of the sequence is represented by ‘1’ or ‘0’ digits, depending on the presence or absence of the structural pattern predefined by F*i*. Previous studies have shown good results by using linear fragments from 5 up to 6 bonds [32], [33]. In order to choose optimal parameters for the compounds present in the HMDB, the ChemAxon parameter optimization criteria was used. From this analysis, the fingerprint length, the maximum fragmentation pattern length and number of bit in the string were 1024, 6, and 2 respectively.

#### Reaction fingerprint

In the case of reaction fingerprints, each compound present in the reaction is represented as a CFP. This fingerprint is a segmented fingerprint that is constituted from 8 chemical fingerprint sections. This reaction fingerprint representation allows us to compare reactions from its structural and transformational features. Parameters were defines as previously stated.

#### Tanimoto coefficient (TC)

The comparison of two fingerprints involves the calculation of the TC. Values of this metric are non-negative numbers. Here, we used the TC dissimilarity (TCdiss) metric. A zero dissimilarity value indicates that the two fingerprints are identical, and the larger the value of the dissimilarity coefficient the higher the difference between the two structures. In its original form, Tanimoto metrics it is a similarity metric (TC_sim_, **Formula 5**):
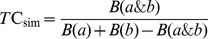
(5)


Where a and b are two binary fingerprints, & denotes binary bit-wise and-operator, | denotes bit-wise or-operator and B(x) is the number of 1 bits in any binary fingerprint x:
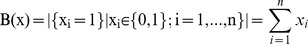



From that it is straightforward to obtain a dissimilarity measure (**Formula 6**):
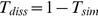
(6)


### Synthetic reaction construction

For synthetic reaction construction, we first defined a set of 374 irreversible generic biochemical reaction operators (BROs) that has been used in previous studies, mostly for prediction purposes for metabolic engineering [51]–[53], enzyme promiscuity analysis [31], [54], and xenobiotics degradation [55]. These BROs can represent approximately 75% of enzymes present in the KEGG Database and 72% of BRENDA EC numbers. Essentially, a BRO is constructed based on the smallest substructure representing the structural changes of substrates and products in a specific reaction. Each BRO is related to specific cofactors and a third-level EC number for further reaction reconstruction and identification. For BRO representation, we used SMIRKS [56], a language used for describing generic reactions (transformations) by using SMARTS [56] representation of the reaction's substructures. A SMARTS pattern may include not only a specification of reaction center, but also a specification of a local structure that must occur or is necessarily absent based on our best understanding of the relevant biochemistry [57]. After BRO construction, we generate all possible reactions that may occur and every compound that may be produced given the previous selected list of human metabolites. Then, specific cofactors were assigned, and for filtering purposes mass balance was performed.

### Compound clustering analysis

For each mutated enzyme, Maximum Common Substructure (MCS) analysis was used in order to identify the most common chemical patter from all predicted products and substrates. The idea is to identify from a range of chemical structures, the largest substructure common in all of them.

For all calculations, with regard to handling compounds, building reaction, substrate fingerprint generation, reaction fingerprint generation, TC dissimilarity calculations, BROs simulations, and MCS analysis we used MATLAB linked with ChemAxon's package libraries, specifically Marvin, JChem Base, Standardizer and Reactor [(ChemAxon, Budapest,Hungary, www.chemaxon.com)].

## Supporting Information

File S1 Full list of 96 recurrently mutated enzyme encoding metabolic genes and their mutation rates.
(XLSX)

File S2 All detailed information of predicted promiscuity reactions for GoF analysis.
(XLSX)

File S3 18 cancer and normal genome-scale metabolic models in SBML.
(ZIP)

Text S1 Supporting results including supporting figures, tables, and text.
(PDF)
