# Supplementary material for: A Systems Approach to Predict Oncometabolites via Context-Specific Genome-Scale Metabolic Networks
Source: PLoS Comput Biol. 2014 Sep 18;10(9):e1003837. doi: 10.1371/journal.pcbi.1003837 (PMC4168981; doi:10.1371/journal.pcbi.1003837)
Supplement: Text S1 — Supporting results including supporting figures, tables, and text. (PDF) [file pcbi.1003837.s004.pdf]

A systems approach to predict oncometabolites via context-specific genome-scale metabolic networks

Hojung Nam[1, 2*], Miguel Campodonico[1, 3], Aarash Bordbar[1], Daniel R. Hyduke[1†], Sangwoo Kim[4], Daniel C. Zielinski[1], Bernhard O. Palsson[1, 5*]

[1]Department of Bioengineering, University of California San Diego, 9500 Gilman Dr., La Jolla, CA, 92093-0412, USA
[2]School of Information & Communications, Gwangju Institute of Science and Technology (GIST), 261 Cheomdan-gwagiro, Buk-gu, Gwangju 500-712, Republic of Korea
[3]Centre for Biotechnology and Bioengineering, CeBiB, University of Chile, Plaza Ercilla 847, Santiago, Chile.
[4]Severance Biomedical Science Institute, Yonsei University College of Medicine, 50-1 Yonsei-ro, Seodaemun-gu, Seoul 120-752, South Korea
[5]Department of Pediatrics, University of California San Diego, 9500 Gilman Dr., La Jolla, CA, 92093, USA
[†]Current address: Department of Biological Engineering, Utah State University, 4105 Old Main Hill, Logan, UT 84322

*** Corresponding authors**
Email: hjnam@gist.ac.kr, palsson@ucsd.edu
Phone: +82-62-715-2641, +1-858-534-5668
Fax: +82-62-715-2204, +1-858-822-3120

Supporting text

Table of Contents

Additional analysis with other TCGA mutation data sets 3
Table S1 3
Figure S1 4
Flowchart representation of significantly mutated enzymes determination and the prediction of oncometabolites 5
Figure S2 5
Mutation rates in previously identified oncometabolite producing genes 6
Table S2 6
Table S3 7
Pathways related to transport showed more frequent transcription alterations in various cancer types and had more frequent mutations 7
Figure S3. 9
Assessment of reconstructed models 10
Table S4 10
Table S5 11
Table S6 11
Figure S4 12
Figure S5 13
Figure S6 14
Reconstructed models accuracy validation 15
Figure S7 15
Figure S8 17
LoF oncometabolite prediction 18
Figure S9 18
Validation of predicted oncometabolites 19
Table S7 19
GoF synthetic reaction reconstruction and filtering pipeline 21
Figure S10 22
GoF mutants and predicted 24 dominant substructures of candidate oncometabolites 23
Figure S11 23
Table S8 24
References 30

Additional analysis with other TCGA mutation data sets

We further analyzed mutations that occur on enzymatic genes using additional TCGA mutation data sets. Here, mutation data sets of four additional cancer types including colon, ovarian, rectal, and uterine were used (**Table S1**). As the TCGA consortium doesn't provide available gene expression data sets for these four cancer types, further analyses including model reconstruction and oncometabolites predictions were not conducted for these cancer types. As Figure S1 shows, we found the results to be qualitatively robust with improvements in the data collection.

Table S1. Additional TCGA mutation data sets.

**Cancer type**<br>**(Histological sub type)**	**Mutation source**<br>**(No. samples)**
Colon<br>(Adenocarcinoma)	TCGA<br>(423)
Ovarian<br>(Serous Cystadenocarcinoma)	TCGA<br>(589)
Rectal<br>(Adenocarcinoma)	TCGA<br>(169)
Uterus<br>(Uterine Corpus Endometrioid Carcinoma)	TCGA<br>(500)

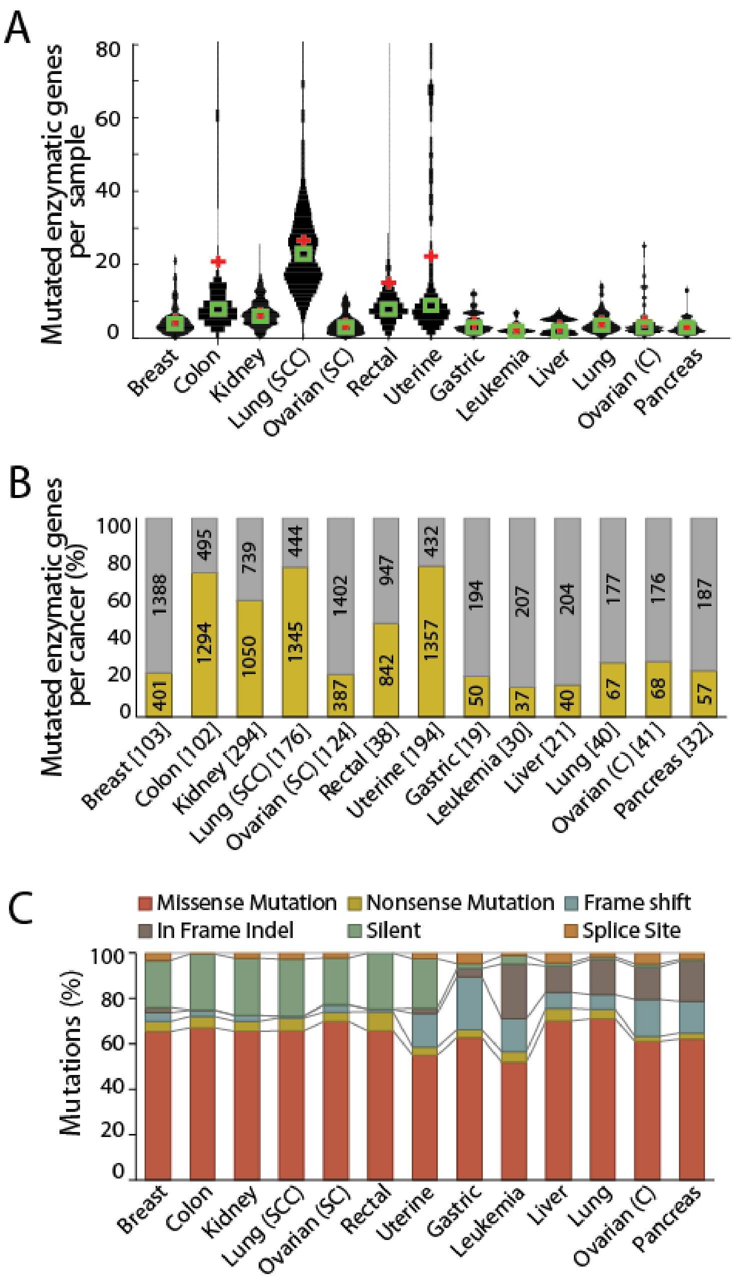

Figure S1. Overall statistics on genetic mutations in metabolic genes. **(A)** The violin plot depicts the distribution of the number of mutated enzymatic genes per patient (sample) in each cancer type (Green box: median, red asterisk: average). In median, 5~20 enzymatic genes were mutated per patient (sample). **(B)** The percentage of mutated enzymatic genes per cancer type. The number of mutated genes per cancer type is equal to the number of unionized mutated enzymatic genes of samples in each cancer type. **(C)** The Percentage of mutation types. Missense mutation was mostly frequently observed across cancer types.

Flowchart representation of significantly mutated enzymes determination and the prediction of oncometabolites

From the 96 recurrently mutated metabolic genes, we next selected genes that could have significant functional impacts on their catalytic activities due to the mutations (**Figure S2A**). First, isoenzymes were filtered out because an unexpected mutational malfunction of one isoenzyme could be substituted by functions of other isoenzymes having duplicated catalytic activities. Second, mutated genes having smaller functional impact were removed. The impact of a mutation in the recurrently mutated genes was assessed by using the functional impact score (FIS) previously proposed [1]. A FIS is derived from multiple sequence alignments of amino acid sequence homologs, thus, the score is based on the evolutionary conservation of a mutated residue in a protein family. Function impact scores (FIS) of mutations on enzymes were calculated from http://mutationassessor.org [1]. By applying the aforementioned criteria, 20 metabolic genes were selected for detailed analysis. These 20 genes are recurrently mutated in samples (≥ 5%), and are expected to have significant functional impact from their genomic sequence mutations (**Figure 3B, File S1**).

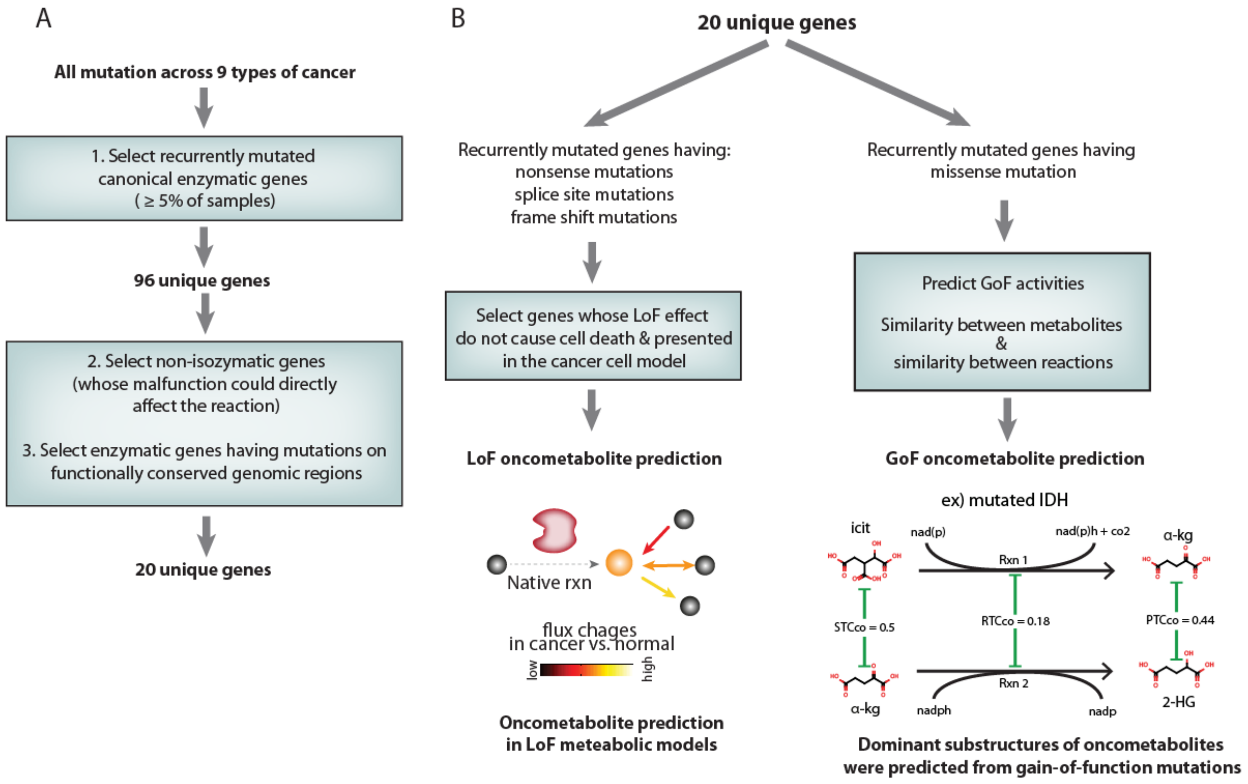

Figure S2. (**A**) Recurrently mutated enzymatic genes selection workflow. (**B**) Loss-of-function and Gain-of-function analysis with the 20 significantly mutated enzymatic genes.

Mutation rates in previously identified oncometabolite producing genes

Previous studies showed that the point mutations in isocitrate dehydrogenase (IDH1, IDH2) confer a new metabolic enzymatic activity that produces an oncometabolite (e.g. 2-hydroxyglutarate (2-HG), from α-ketoglutarate). 2-HG shows a 100-fold increased concentration in glioma and acute myeloid leukemia's (AML) patients with IDH1 or IDH2 missense mutations. This increased concentration of 2-HG competitively inhibits α-ketoglutarate (α-KG) binding to histone demethylases, thus blocking differentiation of cells [2-4]. In parallel to IDH, loss-of-function mutations on succinate dehydrogenase (SDH) and fumarate hydratase (FH) cause the accumulation of succinate and fumarate, respectively, which acts a competitive inhibitor of 2-oxoglutarate-dependent oxygenases that regulate hypoxia-inducible factor (HIF) oncogenic pathway [5-7].

However, in this study, IDH2 / SDHB in gastric and FH in ovarian cancer were found to be mutated in more than 5% of samples (**Table S2**). Also, while previously identified mutations in IDH1 that produce oncometabolites in brain tumors are showing high functional impact scores [8] (**Table S3**), mutations in IDH2 and FH are found to be not functionally important (analysis done by FIS). Thus, in our study, we applied oncometabolite prediction analysis in SDHB only.

Table S2. Mutation percentage of IDH, FH, and SDH genes in nine cancer types.

Gene	breast	kidney(RCC)	lung(SCC)	gastric	leukemia(AML)	liver(HCC)	lung(adenocarcinoma)	ovarian	pancreas
IDH1	0%	1%	1%	0%	0%	0%	0%	2%	0%
IDH2	0%	0%	0%	5%	0%	0%	0%	0%	0%
FH	0%	0%	1%	0%	0%	0%	2%	5%	0%
SDHA	0%	0%	2%	0%	0%	0%	0%	0%	0%
SDHB	0%	0%	1%	5%	0%	0%	2%	2%	0%
SDHC	0%	0%	0%	0%	0%	0%	0%	2%	0%
SDHD	0%	0%	0%	0%	0%	0%	0%	0%	0%

Table S3. Functional impact scores of previously identified IDH1 mutations that produce oncometabolites in brain tumors [8].

Gene	AA variant	Func. Impact	FI score
IDH1	R132H	**high**	4.54
IDH1	R132C	**high**	4.54
IDH1	R132S	**high**	4.54
IDH1	R132G	**high**	4.54
IDH1	R132L	**high**	4.54
IDH1	R132V	**high**	4.54

Pathways related to transport showed more frequent transcription alterations in various cancer types and had more frequent mutations

With the result of P/A calls of enzymatic genes in nine cancer and normal data sets, we evaluated whether the P/A calls are relevant to the cancer specific metabolism. Here, we first evaluated gene-wise P/A alterations in cancer vs. normal across nine cancer types. In this analysis, we confirmed that the P/A alterations in cancer vs. normal of enzymatic genes were not likely consistent across different cancer types (**Figure S3A**), which is accordance with the previous findings that the expression differences of individual genes vary considerably from cancer to cancer [9]. However, when the alterations were evaluated in the level of functional pathways, several pathways related to popular malignancy features showed significant P/A alterations. A pathway is determined as a significantly altered pathway if the pathway has significantly more number of genes with P/A alterations than same number of random sets of genes.

An alteration score of a pathway ($A(p_i)$) is the total number of genes that are expressed in cancer and not expressed in normal or vice versa in a pathway *i*:

$$A(p_i) = \sum_{j=1}^{k} abs\left(PA_{ij}^{cancer} - PA_{ij}^{normal}\right)$$

$$where \begin{cases} PA_{ij} = 1, if\ \ gene\ j\ is\ present\ (expressed) \\ PA_{ij} = 0, if\ \ gene\ j\ is\ absent\ (not\ expressed) \end{cases}$$

Next, to calculate the significance of $A(p_j)$, we used a *P-value* based on the background distribution using 10,000 randomly expression sets for each case:

$$P\text{-}value(p_i) = \Psi_k(\mathrm{A}(\mathrm{p_i}))$$

where $\Psi_k(x)$ is a right-side cumulated probability up to x on the background distribution of a pathway's alteration score with size k. Note that to reduce the bias to expressions and size of pathway, we permutated the real expressions of input data and generated a background distribution for each size of pathway. The basic implication of the scoring function for identifying altered pathways is a statistical test for the null hypothesis, 'observed P/A of gene expression of enzymes of a metabolic pathway is purely random.' Once we obtain the P-values of individual pathways, we adjusted the P-values to control for False Discovery Rate (FDR).

Notably, reactions transporting (exporting, secreting) substances between the inside and outside of cellular space were consistently altered across all types of cancer. Furthermore, we confirmed that these altered pathways were implicated in enzyme mutations. The result showed that enzymes in functional pathways with frequent P/A alterations across nine cancer (cluster 1 in **Figure S3B**) were more often mutated in both of TCGA and CCLE data sets (**Figure S3C-S3E**). This observation leads to a speculation that enzymes having changed catalytic activity due to the missense mutations may provide gene expression alterations in functionally associated reactions. Thus, we can conclude that the P/A alterations of gene expression used in this study represent cancer metabolism that is associated with mutations.

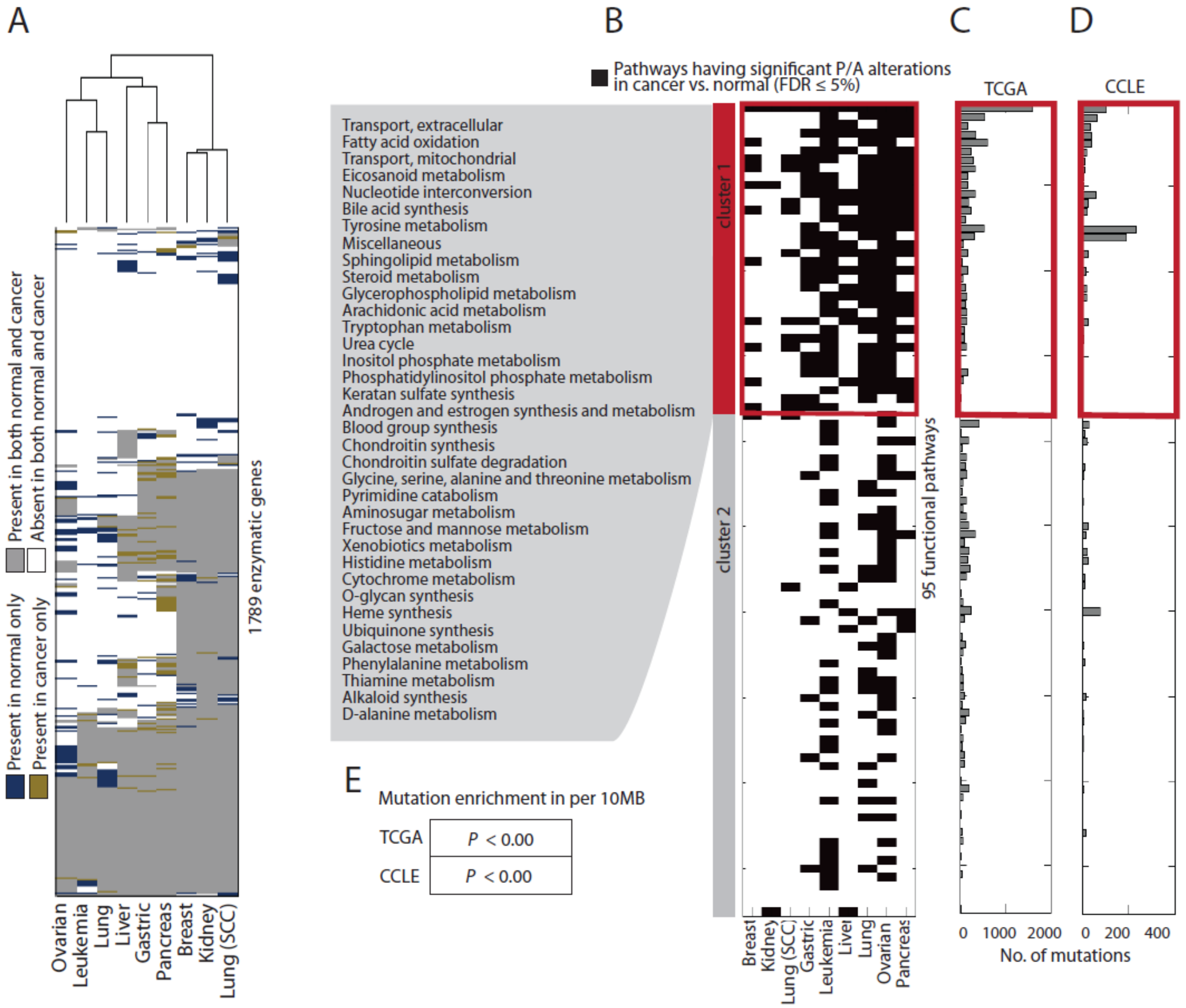

Figure S3. (A) The heatmap depicts alterations of present/absent (P/A) enzymatic genes in cancer vs. normal across nine cancer types. **(B)** The heatmap depicts alterations of metabolic functional pathways across nine cancer types (Black: altered, white: not altered). The pathways were clustered based upon the frequency of alterations across nine cancers (Cluster 1: altered in more or equal to four cancer types, cluster 2: altered in less than four cancer types). The bar-charts next to the heatmap show the number of missense mutations observed in each of the metabolic pathways corresponding to the heatmap shown in B. **C** – the number of mutations in TCGA data sets, **D** – the number of mutations in CCLE data sets. (**E**) The significance of the over representation of the number of mutations in the cluster 1. The table shows P-values from Binomial tests against the distribution of the total length of genes in clusters, respectively.

Assessment of reconstructed models

For the reconstruction of cancer specific and its matched normal metabolic networks, gene expression data sets of cancer and matched normal were used. Here, the specific characteristics of cancer and normal metabolic models were represented by the rerouted network structure based upon the presence or absence of an enzymatic reaction in the intracellular environment of cancer and normal cells. The present/absent (P/A) calls of the Affymetrix gene expression were made using the 'mas5calls' function in the 'affy' package (ver. 1.28.0) implemented in R (ver. 2.15.0), and a threshold of 10 read maps was used to define detection of the P/A calls of RNA-seq data at the gene level [10]. In our study, genes that are expressed in more than 99% of the total number of samples in a data set are finally determined as present (expressed) genes.

Table S4. Vitamin related exchange reactions

Class	Exchange reactions
Vitamin A	‘EX_retfa(e)';'EX_retinol(e)';'EX_retinol_9_cis(e)';'EX_retinol_cis_11(e)';'EX_retn(e)';'EX_11_cis_retfa(e)';'EX_9_cis_retfa(e)'
Vitamin B1	'EX_thm(e)';'EX_thmmp(e)';'EX_thmtp(e)';'EX_thym(e)';'EX_thymd(e)'
Vitamin B2	'EX_ribflv(e)'
Vitamin B3	'EX_nac(e)';'EX_ncam(e)'
Vitamin B6	'EX_pydam(e)';'EX_pydx(e)';'EX_pydxn(e)';'EX_4pyrdx(e)'
Vitamin B9	'EX_5fthf(e)';'EX_5mthf(e)';'EX_5thf(e)';'EX_6dhf(e)';'EX_6thf(e)';'EX_7dhf(e)';'EX_7thf(e)';'EX_dhf(e)';'EX_fol(e)';'EX_thf(e)'
Vitamin C	'EX_ascb_L(e)'
Vitamin D	'EX_2425dhvitd2(e)';'EX_2425dhvitd3(e)';'EX_25hvitd2(e)';'EX_25hvitd3(e)';'EX_vitd2(e)';'EX_vitd3(e)'
Vitamin E	'EX_avite1(e)';'EX_avite2(e)';'EX_bvite(e)'
Vitamin H	'EX_biocyt(e)';'EX_btn(e)'
EFA	'EX_lnlc(e)';'EX_arach(e)'

The P/A call results of gene expression are then incorporated to the reactional space using Gene Inactivity Moderated by Metabolism and Expression (GIMME) algorithm implemented in COBRA Toolbox v2.0 [11,12]. For the medium condition, a standard RPMI-1640 condition was used in all simulation [13,14]. Also, we allowed uptakes to additional vitamins exchange reactions in order to activate pathways (**Table S4**), and constrained models to uptake glucose, oxygen and secrete CO_2, biomass. Finally, nine cancer and normal GEMs are reconstructed (Detailed information of reconstructed models is shown in the **Table 2**).

Most of all reconstructed models produce presumably reasonable uptake and secrete flux states (see flux variation ranges in the **Table S5, S6**). Although several models do not secrete any CO_2 in the optimal flux states, this problem is not a big concern in our study since the representative flux states in our results were determined from the sampling points within the suboptimal solution space (90% of optimality solution space) that is the flux variation ranges shown in **Table S5, S6**.

Table S5. Flux variability ranges of cancer models

	Optimal Flux [min Flux, max Flux] in cancer models			
	O_2	glc	CO_2	BM
Breast	-225.28<br>[-490.03, 0.00]	-5.00<br>[-5.00, 0.00]	77.72<br>[0.00, 217.34]	0.09<br>[0.08, 0.09]
Kidney	-311.52<br>[-523.15, 0.00]	-5.00<br>[-5.00, 0.00]	0.00<br>[0.00, 217.08]	0.09<br>[0.08, 0.09]
Lung (SCC)	-281.80<br>[-523.82, 0.00]	-5.00<br>[-5.00, 0.00]	-0.00<br>[0.00, 225.85]	0.09<br>[0.08, 0.09]
Gastric	-207.02<br>[-413.08, -0.00]	-5.00<br>[-5.00, 0.00]	161.70<br>[0.00, 201.61]	0.08<br>[0.08, 0.08]
Leukemia	-0.27<br>[-15.11, 0.00]	-5.00<br>[-5.00, -1.03]	0.00<br>[0.00, 1.29]	0.08<br>[0.08, 0.08]
Liver	-212.59<br>[-460.86, 0.00]	-5.00<br>[-5.00, 0.00]	21.06<br>[0.00, 203.37]	0.09<br>[0.08, 0.09]
Lung	-5.57<br>[-18.29, -0.00]	-5.00<br>[-5.00, -0.99]	0.00<br>[0.00, 2.86]	0.08<br>[0.08, 0.08]
Ovarian	-11.10<br>[-31.86, -0.24]	-5.00<br>[-5.00, -0.05]	5.06<br>0.00, 5.17]	0.08<br>[0.08, 0.08]
Pancreas	-202.83<br>[-445.23, 0.00]	-5.00<br>[-5.00, 0.00]	0.00<br>[0.00, 208.70]	0.08<br>[0.08, 0.08]

Table S6. Flux variability ranges of normal models

	Optimal Flux [min Flux, max Flux] in normal models			
	O_2	glc	CO_2	BM
Breast	-292.18<br>[-433.23, -177.31]	-5.00<br>[-5.00, 0.00]	0.00<br>[0.00, 228.22]	0.09<br>[0.07, 0.09]
Kidney	-299.05<br>[-443.78, -177.31]	-5.00<br>[-5.00, 0.00]	0.00<br>[0.00, 231.44]	0.09<br>[0.07, 0.09]
Lung (SCC)	-298.33<br>[-445.73, -177.22]	-5.00<br>[-5.00, 0.00]	0.00<br>[0.00, 232.06]	0.09<br>[0.07, 0.09]
Gastric	-258.56<br>[-312.82, -164.77]	-5.00<br>[-5.00, 0.00]	0.00<br>[0.00, 212.74]	0.08<br>[0.07, 0.08]
Leukemia	-216.76<br>[-278.02, -153.56]	-5.00<br>[-5.00, 0.00]	224.17<br>[0.00, 224.74]	0.09<br>[0.07, 0.09]
Liver	-237.13	-5.00	86.41	0.09

	[-354.71, -172.57]	[-5.00, 0.00]	[0.00, 223.53]	[0.07, 0.09]
Lung	-271.41<br>[-363.29, -178.42]	-5.00<br>[-5.00, 0.00]	0.00<br>[0.00, 218.72]	0.08<br>[0.07, 0.08]
Ovarian	-282.91<br>[-367.29, -175.68]	-5.00<br>[-5.00, 0.00]	0.00<br>[0.00, 225.07]	0.09<br>[0.07, 0.09]
Pancreas	-283.07<br>[-381.63, -175.14]	-5.00<br>[-5.00, 0.00]	4.46<br>[0.00, 228.44]	0.09<br>[0.07, 0.09]

Here we can confirm that the activity of reconstructed models varies from one model to other models by observing the difference of the total number of flux carrying reactions (**Figure S4A**), also by observing the difference of present / absent patterns in flux carrying reactions in each model (**Figure S4B**)

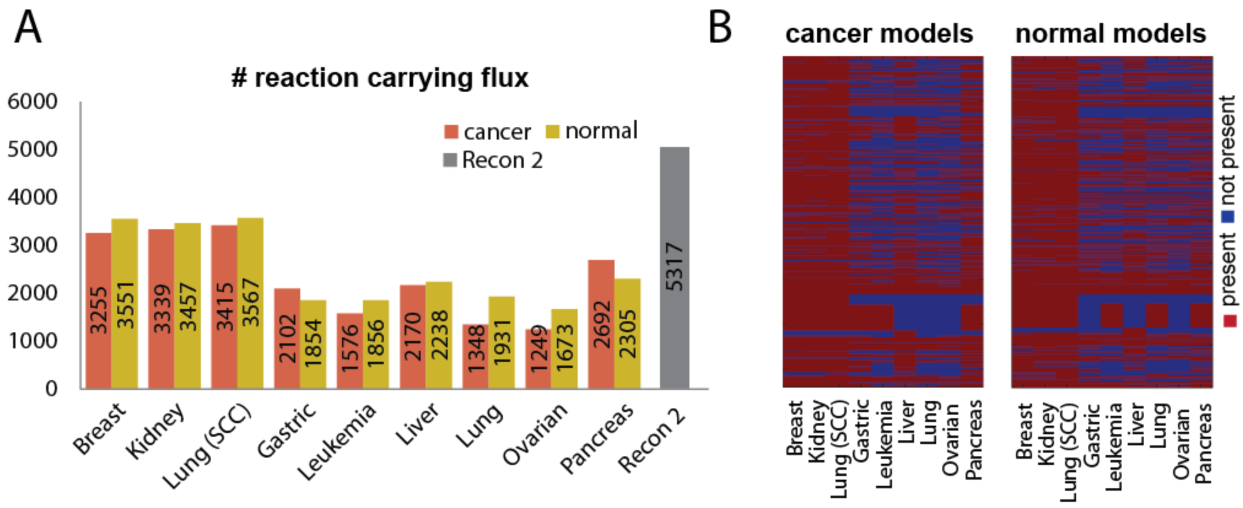

Figure S4. Flux carrying reactions in 18 reconstructed models. **(A)** Each bar depicts the number of flux carrying reactions. Orange: cancer models, Yellow: normal models. Gray: flux carrying reactions in Recon 2. **(B)** The heatmap depicts the differences of flux carrying reactions in 9 cancer models and their matching normal models.

Next, we compared our reconstructed models (reconstructed by GIMME) to the models reconstructed by a different tissue specific model reconstruction method (e.g., mCADRE [15]). We assessed the similarity of models by evaluating the number of overlapping functionally active reactions. When comparing our GEM to the model built by the mCADRE method, we confirmed that high percentages of functionally active reactions (flux carrying reactions) are commonly observed between two models. For example, while the breast cancer model reconstructed by GIMME includes 3255 active reactions, the breast cancer model with mCADRE includes 3722 and the two models share 3010 reactions (**Figure S5**).

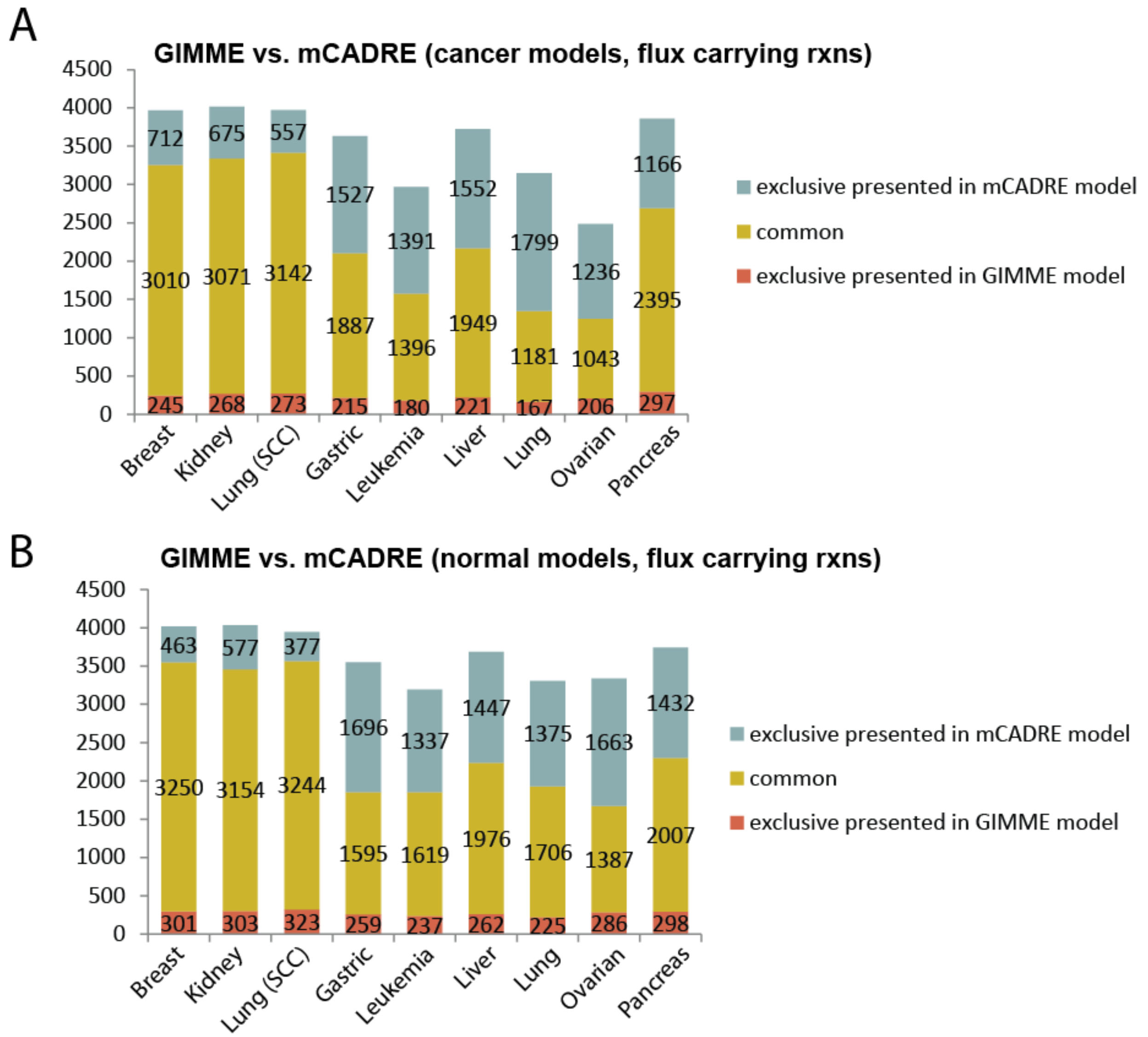

Figure S5. The overlapping of flux carrying reactions in GIMME models and mCADRE models. (**A**) cancer models, (**B**) normal models.

Finally, we compared our reconstructed normal models to the models previously published normal models [16]. Since the models from the Thiele et al. study are reconstructed by using protein expression data sets that have smaller coverage compare to genome-wide gene expression data sets, the size of the models from the Thiele et al. study is smaller than our model. However, we can observe significant number of overlapping reactions between two models (**Figure S6A**). The result was consistent when comparing overlapping reactions in gene associated reactions as well (**Figure S6B**).

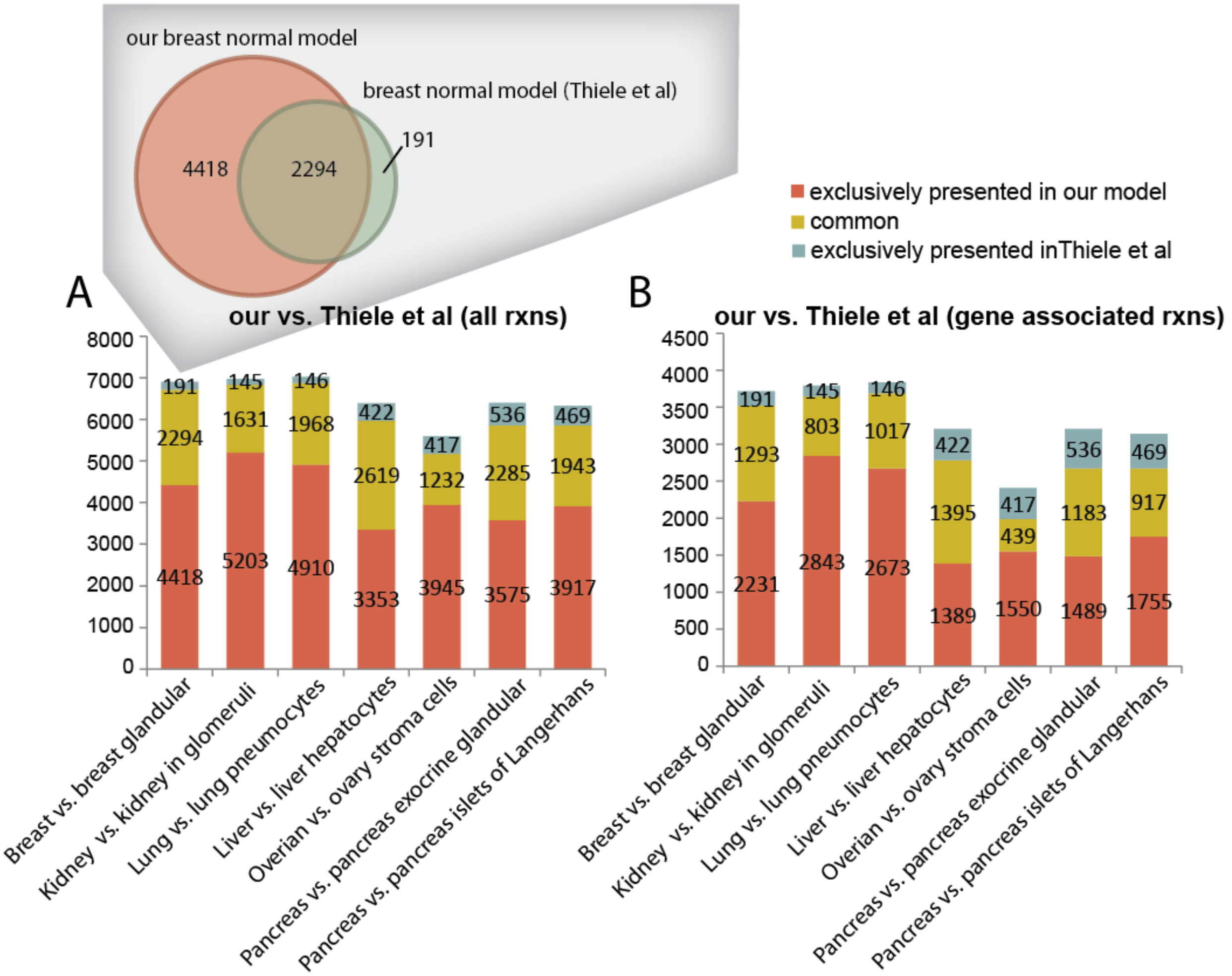

Figure S6. The number of overlapping reactions between our reconstructed model and a model in Thiele et al.. **(A)** For all reactions. **(B)** For gene associated reactions.

Reconstructed models accuracy validation

The correlation between gene expression and reactions of each model was determined by correlations of two vectors of Pearson's correlation coefficient (PCC) calculated from pairwise correlations of P/A of gene expression and pairwise correlations of presence/absence of reactions between cancer types (**Figure 4A**). Here, we note that high PCC values of gene expression do not necessarily represent high correlation of gene expression ratio as the PCC values represent similarities of present-absent call results of expression of metabolic genes. Thus, the cancer cells which showed high correlation could have significant differences in expression of regulatory genes (since regulatory genes are not included in genome-scale metabolic models), and even could have significant gene expression fold-changes in metabolic genes.

Values of the final correlation coefficients varied from 0.90 to 0.98. Also, the significance of the final PCC values were determined by comparing PCC values among 10,000 set of random PCC values generated from permuted gene expression P / A call (**Figure S7**).

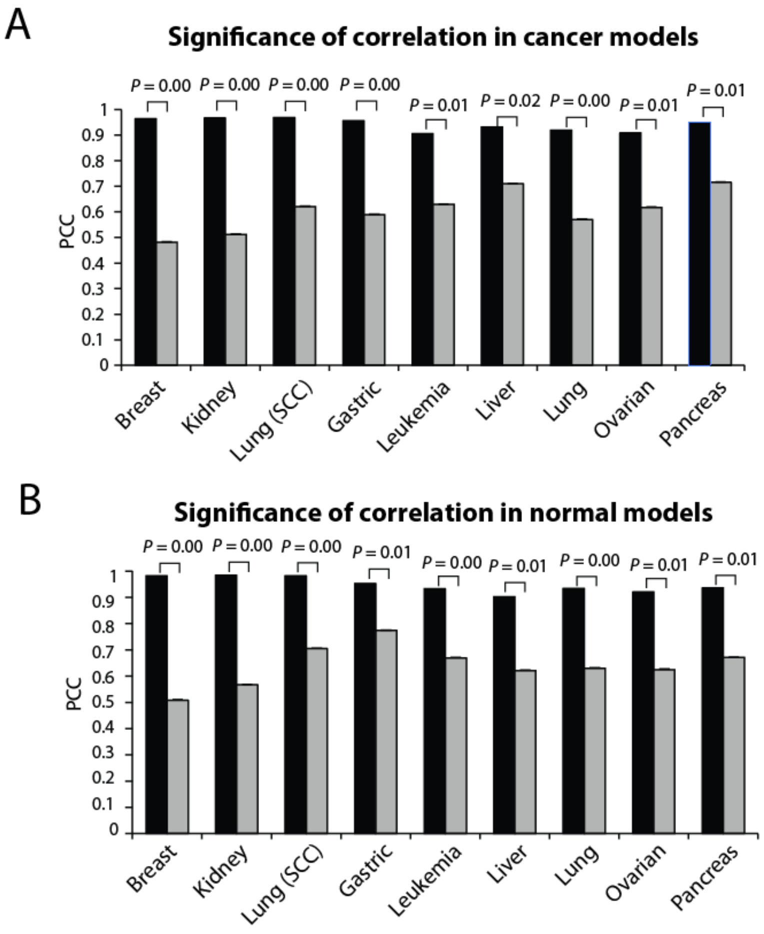

Figure S7. Correlation between gene expression P/A calls and the network structure for each cancer type. The black bar depicts the Pearson's correlation coefficient (PCC) of the reconstructed

models. The grey bar denotes the median value of 10,000 PCC values from the randomly permuted gene expression P/A calls. The error bar shows the standard error mean (SEM) of 10,000 random PCC values. **(A), (B)** PCC value and its significance in cancer and normal model, respectively.

Next, we tested the accuracy of the simulated flux states of the reconstructed models by evaluating whether the simulated flux state correctly predicts metabolic states of cancer and normal cells. To address the question of whether model predictions are consistent with gene expression changes, all significantly changed fluxes are then decomposed into a list of genes that help to catalyze the reactions using the gene-protein-reaction associations in the model. Through this, one can obtain lists of genes that are expected to be up-regulated or down-regulated (genes that are associated both with reactions that increase and other reactions that decrease are removed from the analysis since the kinetics for the different reactions are often not known). The main hypothesis here is that if the flux through a network is predicted to increase (or decrease) in magnitude, that the expression level will increase (or decrease) to meet the change in flux. These model-predicted up- and down-regulated genes can be compared to gene expression data for different growth conditions.

Figure 4B shows the accuracy of the predictions for the significantly changed flux (P-value < 0.001, fold-change ≥ 2). The results were found to be fairly accurate. Among the nine cancer models, eight cancer types, except the kidney cancer, showed better accuracy than random tests. Liver, lung and pancreas cancer achieved significantly accurate results (P-value < 0.01, Random tests). Furthermore, our results were qualitatively robust with variations in the P-value and fold-change thresholds (**Figure S8**)

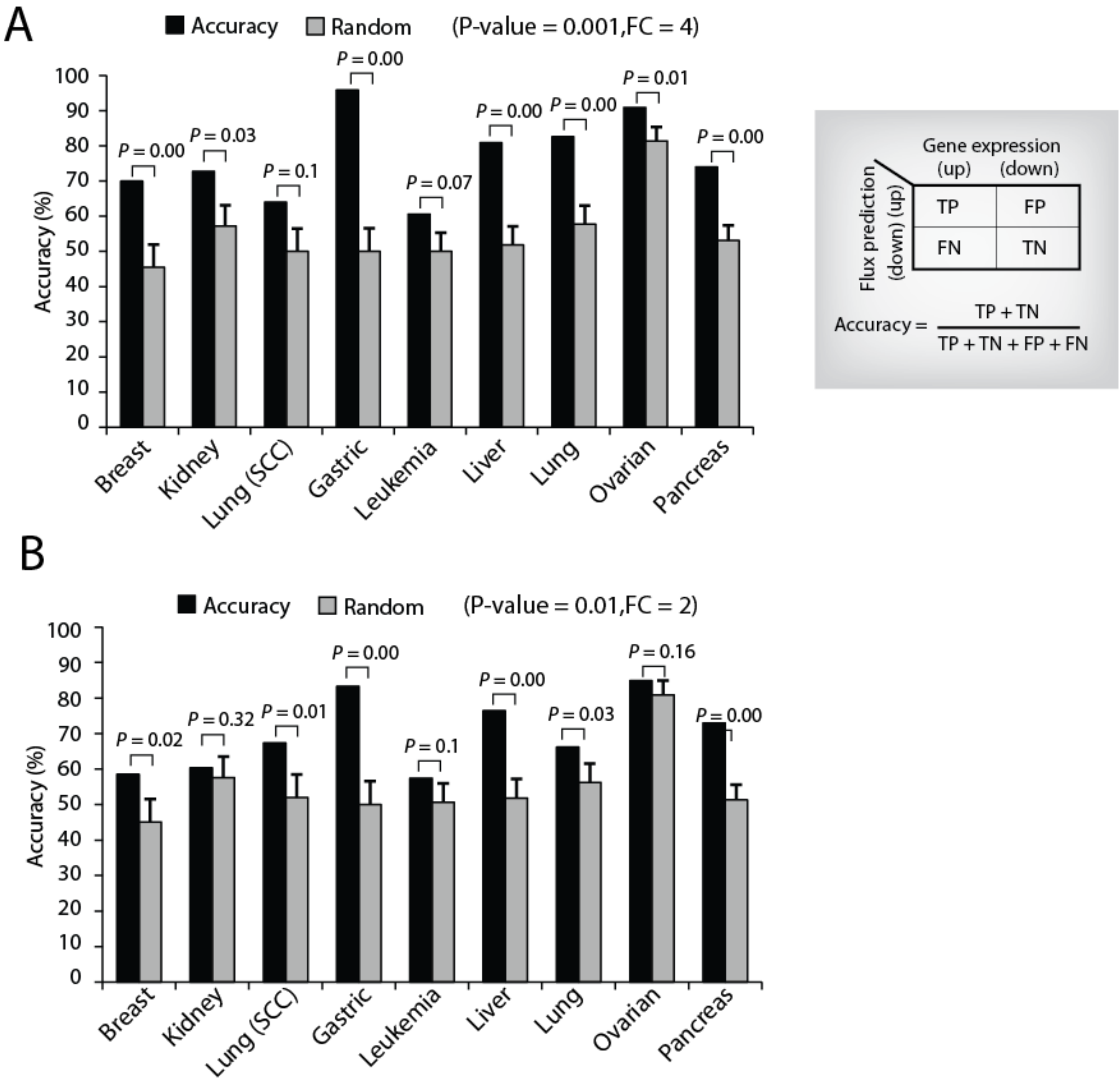

Figure S8. The prediction of gene expression changes from changes in flux was tested against gene expression data sets for cancer vs. normal. The black bar depicts the accuracy of the flux prediction from the reconstructed models. The grey bar denotes the median value of 10,000 accuracies from the random flux. The error bar shows the standard error mean (SEM) of 10,000 random accuracies. **(A)** P-value = 0.001 and Fold change = 4 threshold. **(B)** P-value = 0.01and Fold change = 2 threshold.

LoF oncometabolite prediction

To predict LoF oncometabolites, we modified a reconstructed cancer model into mutated enzyme deficient models. For example, as shown in Figure 3B, FASN, ACACB, and CAD genes were found to be recurrently mutated in leukemia, thus, each of the FANS-deficient, ACACB-deficient, and CAD-deficient leukemia models were separately built by knocking out the mutated genes one at a time. When applying this process to nine genes that have potential LoF effects, some of the deficient models were excluded since they could not have feasible flux solution state (marked as NF in the **Figure S9**). Also, if the initial cancer models do not include the LoF mutated enzymatic reactions, the deficient model was not considered for further LoF analysis (marked as NP). Thus, finally, LoF analyses were done on 13 deficient models (**Figure S9**).

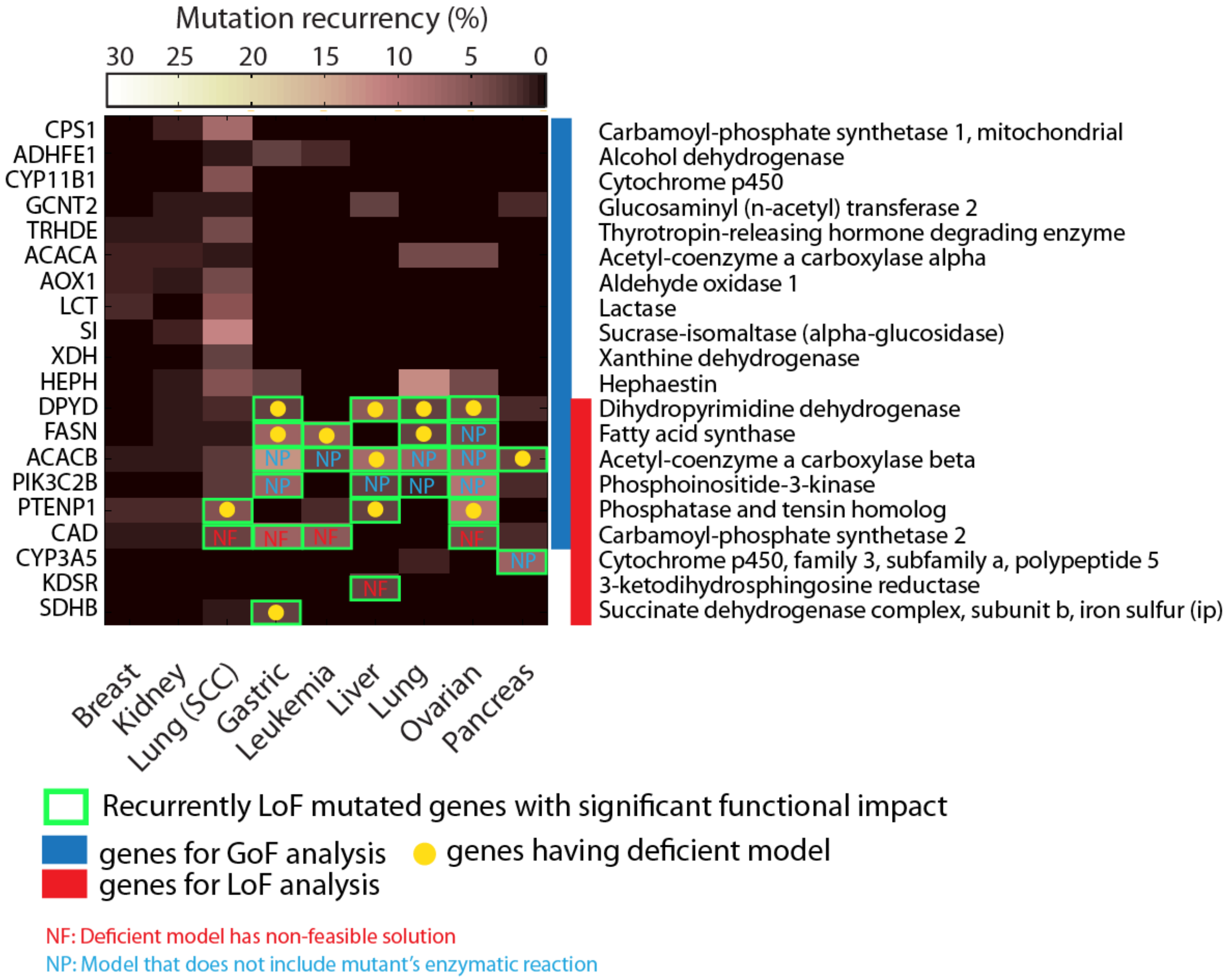

Figure S9. The selected 20 metabolic genes that are recurrently mutated in samples (≥ 5%), and are expected to have a significant functional impact from their genomic sequence mutations in any of nine cancer types. Blue box: recurrently mutated genes for GoF analysis (gene having missense

mutations). Red box: recurrently mutated genes for LoF analysis (genes having nonsense, frame shift indels, splice site indels&SNP mutations). Yellow circle: recurrently mutated genes having deficient model, NF: Deficient model that has no feasible solution, NP: Model that does not include mutant's enzymatic reaction.

Validation of predicted oncometabolites

In this study, 15 unique metabolites catalyzed by the mutated enzymes and surrounded by significantly changing flux were predicted as context-specific loss-of-function (LoF) oncometabolites (**Figure 5A**), and 24 dominant substructures of candidate oncometabolites are predicted as a result of gain-of-function (GoF) mutations (**Figure 5B**).

First, we checked whether previously known oncometabolites (2-HG, succinate, fumarate) were detected in our prediction. As a result, we found that succinate and fumarate were predicted as LoF oncometabolites in the SDHB deficient gastric cancer (**Figure 5A**).

Second, the relevance of our predicted oncometabolites to cancer is tested using literature evidence (information gathered from Human Metabolome DB (HMDB)) [17]). As a result, among 15 predicted LoF oncometabolites, many of metabolites were found to be associated with human diseases but no metabolite was found to be related to cancer in previous studies (**Table S7**).

Table S7. Predicted oncometabolites and their detailed information.

**Name**	**Chemical Formula**	**Average Molecular Weight**	**Cellular locations**	**Associated Disorders and Diseases (OMIM ID)**
3,4-Dihydroxymandelaldehyde	C8H8O4	168.1467	Cytoplasm	N/A
3,4-Dihydroxymandelate	C8H8O5	184.1461	Cytoplasm	N/A
(4-hydroxy-3-methoxyphenyl)acetaldehyde	C9H10O3	166.1739	Cytoplasm	N/A
5,6-dihydrothymine	C5H8N2O2	128.1292	Cytoplasm	Dihydropyrimidinase deficiency (222748);<br>Beta-ureidopropionase deficiency (613161)
5,6-dihydrouracil	C4H6N2O2	114.1026	Cytoplasm;<br>Nucleus	Dihydropyrimidine dehydrogenase deficiency (274270);<br>Hypertension (145500)
Adenosine	C10H13N5O4	267.2413	Extracellular;	Septic shock (N/A)

			Mitochondria; Lysosome	
Fumarate	$C_4H_4O_4$	116.0722	Extracellular; Membrane; Mitochondria	Alzheimer's disease (104300); Lung Cancer (211980); Fumarase deficiency (606812)
D-glucose	$C_6H_{12}O_6$	180.1559	Extracellular; Lysosome; Endoplasmic reticulum; Golgi apparatus	Alzheimer's disease (104300) Diabetes mellitus type 2 (125853) Growth hormone deficiency (139250) Acute myelogenous leukemia (602439) Hyperlipidemia (238600) 3-methyl-crotonyl-glycinuria (210200) Addison's Disease (240200)
Palmitate	$C_{16}H_{32}O_2$	256.4241	Cytoplasm; Extracellular; Membrane (predicted from logP); Endoplasmic reticulum; Peroxisome	N/A
Homovanillate	$C_9H_{10}O_4$	182.1733	Cytoplasm	Hypothyroidism (218700); Narcolepsy (161400); Growth hormone deficiency (139250); Schizophrenia (181500); Panic disorder (167870); Hereditary spastic paraplegia (182601); Autism (209850); Parkinson's disease (168600); Friedreich's ataxia (229300); Major depressive disorder (608516); Aromatic L-amino acid decarboxylase deficiency (107930);
1-phosphatidyl-1D-myo-inositol 3,4,5-trisphosphate(7-)	$C_{11}H_{13}O_{22}P_4R_2$	621.10280	N/A	N/A
phosphatidylinositol 4,5-bisphosphate	$C_{11}H_{19}O_{19}P_3R_2$	548.17840	N/A	N/A
Succinate	$C_4H_6O_4$	118.088	Extracellular;	Canavan disease (271900);

			Mitochondria; Endoplasmic ;reticulum; Peroxisome	Alzheimer's disease (104300); Lung Cancer (211980)
Thymine	C5H6N2O2	126.1133	Extracellular	Beta-ureidopropionase deficiency (613161); Dihydropyrimidine dehydrogenase deficiency (274270)
Uracil	C4H4N2O2	112.0868	Extracellular	Canavan disease (271900); Argininemia (207800); Carbamoyl Phosphate Synthetase Deficiency (237300); Dihydropyrimidine dehydrogenase deficiency (274270); Hypertension (145500)

GoF synthetic reaction reconstruction and filtering pipeline

The developed framework for GoF synthetic reaction reconstruction and filtering, involves three mayor steps. First, the set of human metabolites for synthetic reaction construction was obtained from The Human Metabolome Database (HMDB) [17]. The HMDB was filtered due to the large amount of metabolites present in the database that makes the synthetic reaction construction procedure intractable in terms of computational time. This was performed by using chemical fingerprint (CFP), where a dissimilarity Tanimoto coefficient (TC) matrix was calculated between each substrates and products of mutant enzymatic reactions and each metabolite present in the HMDB. Then, by using different TC cutoffs values (0.8 – 0.005), the number of HMDB metabolites to feed the synthetic reaction calculation for Biochemical Reaction Operators (BROs) simulation was determine. For each cutoffs values, HMDB metabolites scores of less than or equal to the cutoff values were counted. Finally, in order to determine the set of metabolites and constraint the computational time for further synthetic reaction construction and analysis, cutoff values were set for each mutated enzyme reaction (**File S2**). The compound list for BROs simulation was reduced from 40,251 to 552 metabolites in total. Second, by using a predefined set of BROs, all possible reactions that may occur and every compound that may be produced given the previous set of human metabolites were generated. In total more than 200,000 synthetic reactions were generated.

Finally, in order to predict promiscuous reactions of GoF mutants, synthetic reactions were compared and filtered for each GoF mutant enzyme. Based on the IDH GoF mutation case, different TC cutoff values were determined as shown in Figure S10. In this case, 5 metrics were chosen for reaction comparison and filtering. As shown in Figure S10 Rxn 1 corresponds to the native reaction and Rxn 2 represents the enzyme gain of function reaction. In order to characterize the difference between metabolites and cofactors, two different pairs of "pseudo" reactions were defined. PsRrxn

1 and 2 (**Figure S10 B**) were respectively build from Rxn 1 and Rxn2. In this case only substrates and products were used to form both reactions. Same procedure was used in order to construct PsCrxn 1 and 2, but in this case only cofactors were taken into account to construct the "pseudo" reactions. From these pairs of reaction different metrics where calculated in order to numerically characterizes the chemical changes in the reactions due to mutation. As mentin above, these metrics were use as threshold values for filtering mutated enzyme reactions and computationally generated reactions. For the pair of reactions outlined in Figure 10 SA, the substrate Tanimoto coefficient (STCco) and the reaction Tanimoto coefficient (RTCco) were used for filtering. For "pseudo" reactions presented in B only the reaction Tanimoto coefficient (PsRTC) was taken into account. And finally for the las pair of "pseudo" reactions (C), the substrates Tanimoto coefficient (PsTCsub) and the product Tanimoto coefficient (PsCTpro) were choose valid thresholds. According to the later, reactions with scores of less than or equal to the cutoff values were chosen for further substructure oncometabolite reactions)

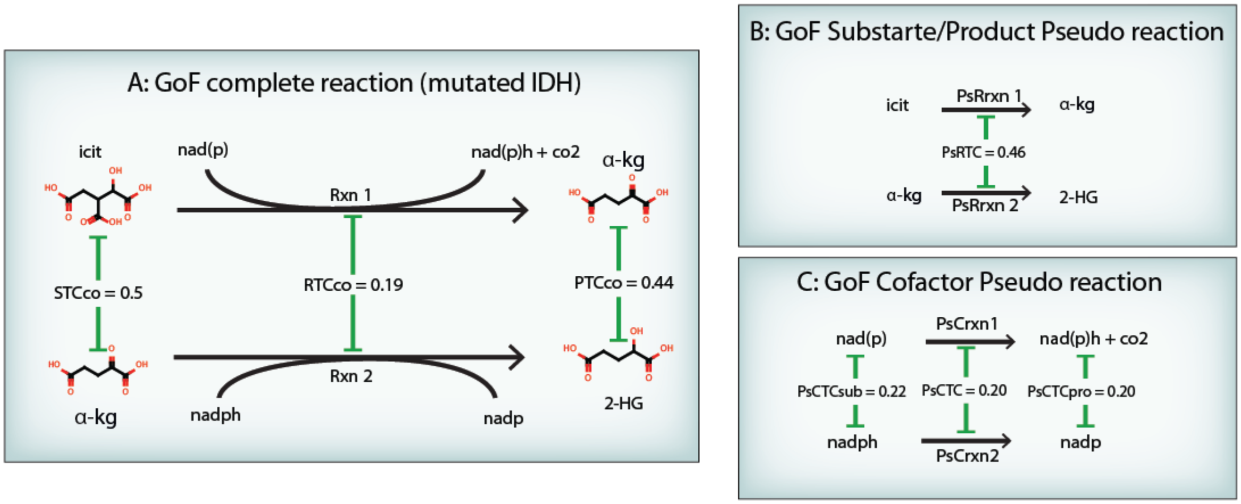

Figure S10. Cutoff values determinations for synthetic reaction construction based on the ODH GoF mutation case. (**A**) Gain-of-function complete reaction metrics definition. (**B**) Gain-of-function metrics based on the substrates and produts. (**C**) Gain-of-function metrics based on the cofactors present in both reactions.

GoF mutants and predicted 24 dominant substructures of candidate oncometabolites

In this study, we used a chemoinformatics approach to predict promiscuous catalytic activities of enzymes resulting from their GoF mutations. Here we assume that a GoF mutant enzyme should be transcribed into RNA in order to have catalytic activities, thus we used gene expression present/absent call result to determine the expression of mutations. Thus, following this criteria, we finally obtained seven genes (HEPE, DPYD, FASN, ACACB, PIK3C2B, PTENP1, and CAD) for the GoF analysis among 17 GoF recurrently mutated genes (**Figure S11**).

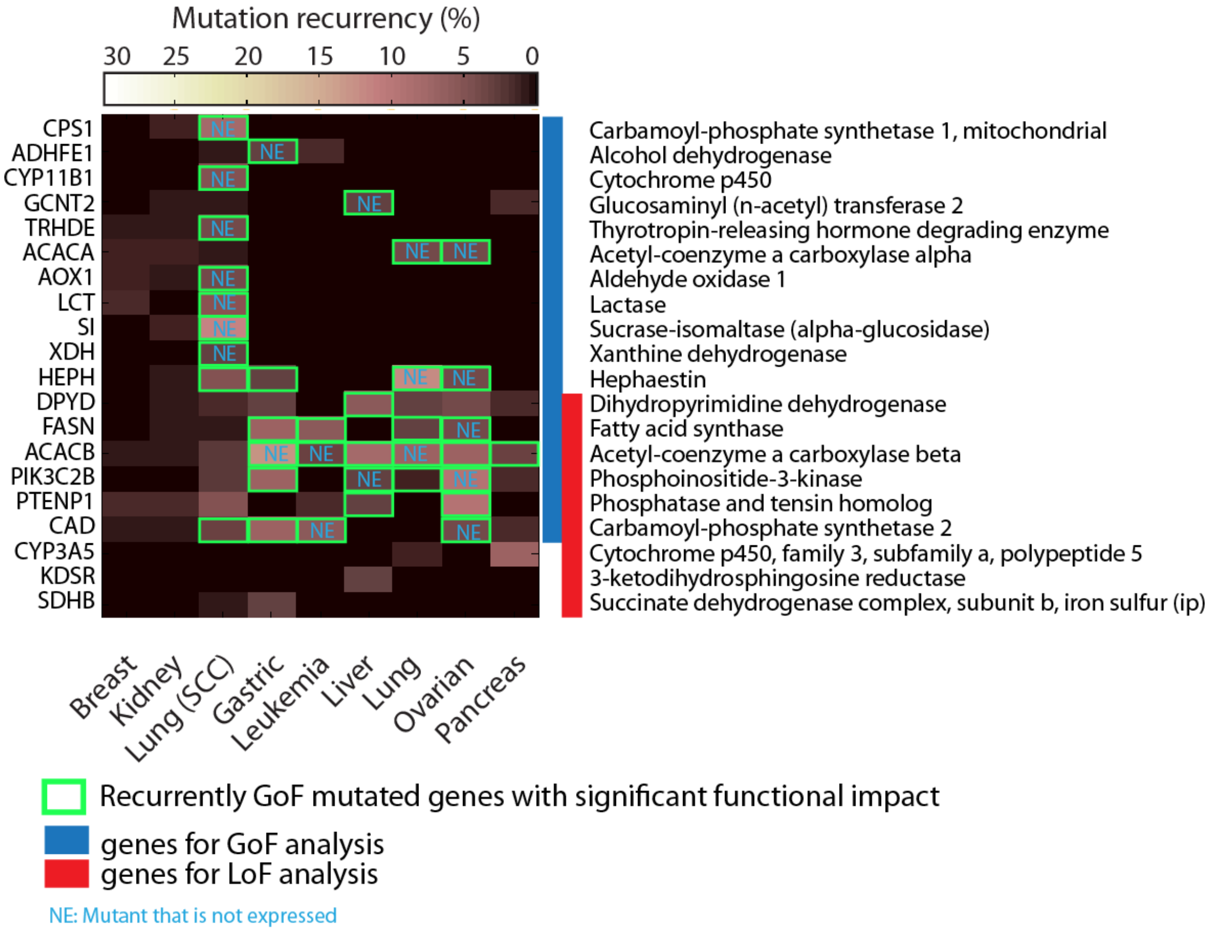

Figure S11. The selected 20 metabolic genes that are recurrently mutated in samples (≥ 5%), and are expected to have a significant functional impact from their genomic sequence mutations in any of nine cancer types. Blue box: recurrently mutated genes for GoF analysis (gene having missense mutations). Red box: recurrently mutated genes for LoF analysis (genes having nonsense, frame shift indels, splice site indels&SNP mutations). NE: mutated gene that is not expressed (not transcribed into RNA).

A summary of mutated enzymes associated reactions and their predicted promiscuity catalytic activities is given in Table S8. For example, for the CAD gene which catalyzing L-glutamine, 2866 potential promiscuity catalytic reactions associated with 170 substrates and 1644 products were predicted in CAD mutant lung (SCC) cancer and gastric cancer (**Figure 5B**, all detailed information of predicted promiscuity reactions is shown in the **File S2**).

We conducted compound similarity analysis in order to demonstrate dominant substructures of promiscuous substrates and products (**Table S8**). The compound structure shown in the Table S8 is the dominantly observed substructure of promiscuous substrates and products. Notable, several promiscuous substrates and products did not have dominant substrates. Finally, we identified 24 promiscuous compound substructures as features of GoF oncometabolites (**Figure 5B**).

Table S8. Mutated enzymes associated synthetic promiscuous reactions and their dominant substructures of potential promiscuity oncometabolites

Gene (Entrez ID)	Native reaction	No. of predicted promiscuous reactions	No. of predicted substrate, dominant substructure	No. of predicted product, dominant substructure
ACACB (32)	ATP(4-) + Bicarbonate + Acetyl-CoA -> ADP + proton + hydrogenphosphate + Malonyl-CoA	25	4<br>N/A	23<br>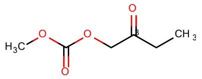
ACACB (32)	Acetyl-CoA + ATP(4-) + Bicarbonate -> proton + ADP + malonyl-CoA(4-) + hydrogenphosphate	25	4<br>N/A	23<br>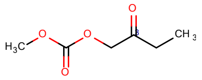
CAD (790)	L-aspartate(1-) + Carbamoyl phosphate <=> proton + hydrogenphosphate + N-Carbamoyl-L-aspartate	21	12<br>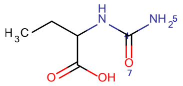	21<br>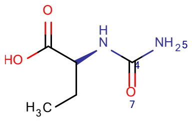
CAD (790)	H2O + 2 ATP(4-) + Bicarbonate + L-glutamine -> 2 ADP + 2 proton + hydrogenphosphate + L-glutamate(1-) + Carbamoyl phosphate	2886	170<br>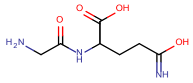	1644<br>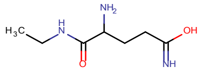
DPYD (1806)	Nicotinamide adenine dinucleotide phosphate + 5,6-dihydrouracil <=> proton +	13	8<br>N/A	2<br>N/A

	Nicotinamide adenine dinucleotide phosphate - reduced + Uracil			
DPYD (1806)	Nicotinamide adenine dinucleotide phosphate + 5,6-dihydrothymine <=> proton + Nicotinamide adenine dinucleotide phosphate - reduced + Thymine	25	12 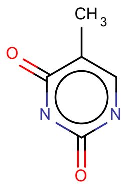	4 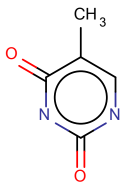
FASN (2194)	Acetyl-CoA + acyl carrier protein <=> Coenzyme A + Acetyl-ACP	2542	490 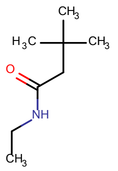	491 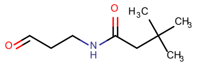
FASN (2194)	3 proton + 2 Nicotinamide adenine dinucleotide phosphate - reduced + Malonyl-CoA + Octanoyl-CoA (n-C8:0CoA) -> H2O + 2 Nicotinamide adenine dinucleotide phosphate + CO2 + Coenzyme A + Decanoyl-CoA (n-C10:0CoA)	822	303 N/A	311 N/A
FASN (2194)	3 proton + 2 Nicotinamide adenine dinucleotide phosphate - reduced + Malonyl-CoA + Decanoyl-CoA (n-C10:0CoA) -> H2O + 2 Nicotinamide adenine dinucleotide phosphate + CO2 + Coenzyme A + Dodecanoyl-CoA (n-C12:0CoA)	822	303 N/A	311 N/A
FASN (2194)	3 proton + 2 Nicotinamide adenine dinucleotide phosphate - reduced + Malonyl-CoA + Dodecanoyl-CoA (n-C12:0CoA) -> H2O + 2 Nicotinamide adenine dinucleotide phosphate + CO2 + Coenzyme A + Tetradecanoyl-CoA (n-C14:0CoA)	822	303 N/A	311 N/A
FASN (2194)	3 proton + 2 Nicotinamide adenine dinucleotide phosphate - reduced + Malonyl-CoA +	822	303 N/A	311 N/A

	Tetradecanoyl-CoA (n-C14:0CoA) -> H2O + 2 Nicotinamide adenine dinucleotide phosphate + CO2 + Coenzyme A + Palmitoyl-CoA (n-C16:0CoA)			
FASN (2194)	3 proton + 2 Nicotinamide adenine dinucleotide phosphate - reduced + Malonyl-CoA + Palmitoyl-CoA (n-C16:0CoA) -> H2O + 2 Nicotinamide adenine dinucleotide phosphate + CO2 + Coenzyme A + Stearoyl-CoA (n-C18:0CoA)	822	303<br>N/A	311<br>N/A
FASN (2194)	9 proton + 6 Nicotinamide adenine dinucleotide phosphate - reduced + Acetyl-CoA + 3 Malonyl-CoA -> 3 H2O + 6 Nicotinamide adenine dinucleotide phosphate + 3 CO2 + 3 Coenzyme A + Octanoyl-CoA (n-C8:0CoA)	777	311<br>N/A	301<br>N/A
FASN (2194)	20 proton + 14 Nicotinamide adenine dinucleotide phosphate - reduced + Acetyl-CoA + 7 Malonyl-CoA -> 6 H2O + 14 Nicotinamide adenine dinucleotide phosphate + 7 CO2 + 8 Coenzyme A + palmitate	777	311<br>N/A	301<br>N/A
FASN (2194)	Malonyl-CoA + acyl carrier protein <=> Coenzyme A + Malonyl-[acyl-carrier protein]	1860	477<br>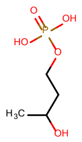	436<br>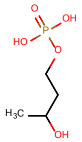
FASN (2194)	Nicotinamide adenine dinucleotide + (S)-3-Hydroxydodecanoyl-CoA -> proton + Nicotinamide adenine dinucleotide - reduced + 3-Oxododecanoyl-CoA	782	254<br>N/A	285<br>N/A
FASN (2194)	Nicotinamide adenine dinucleotide + 5beta-cholestane-	2565	630	681

	3alpha,7alpha,26-triol <=> proton + Nicotinamide adenine dinucleotide - reduced + 3alpha,7alpha-dihydroxy-5beta-cholestan-26-al		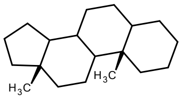	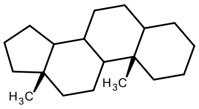
FASN (2194)	O2 + proton + Nicotinamide adenine dinucleotide phosphate - reduced + 5beta-cholestane-3alpha,7alpha,12alpha-triol <=> H2O + Nicotinamide adenine dinucleotide phosphate + 5beta-cholestane-3alpha,7alpha,12alpha,26-tetrol	4688	944<br>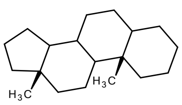	636<br>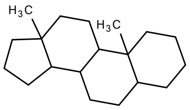
FASN (2194)	Nicotinamide adenine dinucleotide + 3alpha,7alpha,12alpha,24-Tetrahydroxy-5beta-cholestanoyl-CoA -> proton + Nicotinamide adenine dinucleotide - reduced + 3alpha,7alpha,12alpha-trihydroxy-24-oxo-5beta-cholestan-26-oyl-CoA(4-)	749	222<br>N/A	285<br>N/A
FASN (2194)	Nicotinamide adenine dinucleotide phosphate + 5beta-cholestane-3alpha,7alpha-diol <=> proton + Nicotinamide adenine dinucleotide phosphate - reduced + 7alpha-hydroxy-5beta-cholestan-3-one	3985	929<br>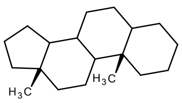	643<br>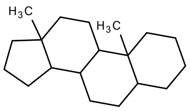
FASN (2194)	Nicotinamide adenine dinucleotide phosphate + 5beta-cholestane-3alpha,7alpha,12alpha-triol <=> proton + Nicotinamide adenine dinucleotide phosphate - reduced + 7alpha,12alpha-dihydroxy-5beta-cholestan-3-one	3726	859<br>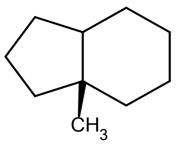	656<br>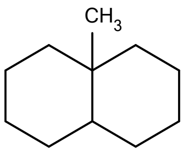
FASN (2194)	H2O + Nicotinamide adenine dinucleotide phosphate + 3,4-Dihydroxymandelaldehyde <=> 2	1033	173	218

	proton + Nicotinamide adenine dinucleotide phosphate - reduced + 3,4-Dihydroxymandelate		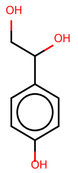	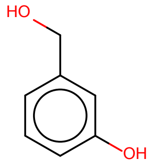
FASN (2194)	H2O + Nicotinamide adenine dinucleotide + (4-hydroxy-3-methoxyphenyl)acetaldehyde <=> 2 proton + Nicotinamide adenine dinucleotide - reduced + Homovanillate	1146	274 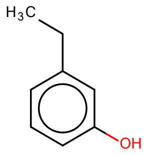	260 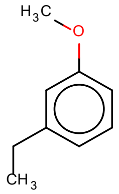
FASN (2194)	Nicotinamide adenine dinucleotide phosphate + D-3-Hydroxyhexanoyl-ACP <=> proton + Nicotinamide adenine dinucleotide phosphate - reduced + 3-Oxohexanoyl-ACP	65	17 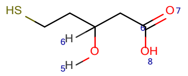	16 N/A
PIK3C2B (5287)	ATP(4-) + 1-phosphatidyl-1D-myo-inositol 4-phosphate(3-) -> ADP + proton + 1-phosphatidyl-1D-myo-inositol 3,4-bisphosphate(5-)	542	217 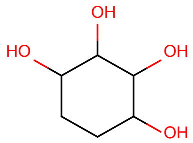	224 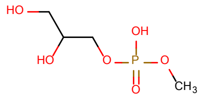
PIK3C2B (5287)	ATP(4-) + 1-phosphatidyl-1D-myo-inositol 4-phosphate(3-) -> ADP(3-) + proton + 1-phosphatidyl-1D-myo-inositol 3,4-bisphosphate(5-)	542	217 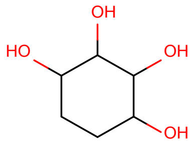	224 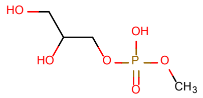
PIK3C2B (5287)	ATP(4-) + 1-phosphatidyl-1D-myo-inositol 5-phosphate(3-) -> ADP + proton + 1-phosphatidyl-1D-myo-inositol 3,5-bisphosphate(5-)	559	216 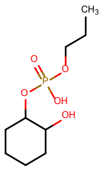	242 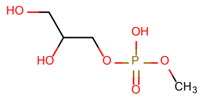
PIK3C2B (5287)	ATP(4-) + 1-phosphatidyl-1D-myo-inositol 5-phosphate(3-) -> ADP(3-) + proton + 1-phosphatidyl-1D-myo-inositol 3,5-bisphosphate(5-)	559	216	242 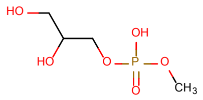

			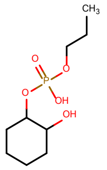	
PIK3C2B (5287)	ATP(4-) + 1-phosphatidyl-1D-myo-inositol(1-) -> ADP + proton + 1-phosphatidyl-1D-myo-inositol 3-phosphate(3-)	407	169 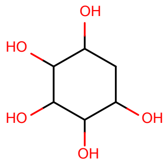	169 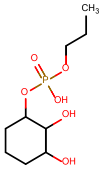
PIK3C2B (5287)	ATP(4-) + 1-phosphatidyl-1D-myo-inositol(1-) -> ADP(3-) + proton + 1-phosphatidyl-1D-myo-inositol 3-phosphate(3-)	407	169 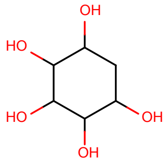	169 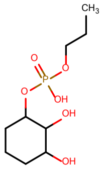
PIK3C2B (5287)	ATP(4-) + 1-phosphatidyl-1D-myo-inositol 5-phosphate(3-) <=> ADP + proton + 1-phosphatidyl-1D-myo-inositol 3,5-bisphosphate(5-)	1071	227 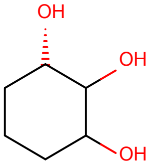	242 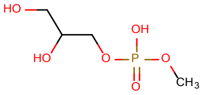
PTENP1 (5728)	H2O + 1-phosphatidyl-1D-myo-inositol 3,4,5-trisphosphate(7-) -> hydrogenphosphate + phosphatidylinositol 4,5-bisphosphate	5	5 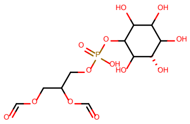	1 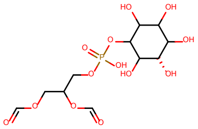
PTENP1 (5728)	H2O + 1-phosphatidyl-1D-myo-inositol 3,4,5-trisphosphate(7-) -> hydrogenphosphate + phosphatidylinositol 4,5-bisphosphate	5	5 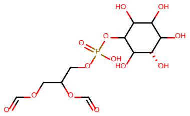	1 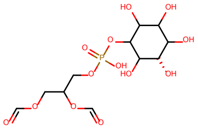

References

1. Reva B, Antipin Y, Sander C (2011) Predicting the functional impact of protein mutations: application to cancer genomics. Nucleic Acids Res 39: e118.
2. Xu W, Yang H, Liu Y, Yang Y, Wang P, et al. (2011) Oncometabolite 2-hydroxyglutarate is a competitive inhibitor of alpha-ketoglutarate-dependent dioxygenases. Cancer Cell 19: 17-30.
3. Lu C, Ward PS, Kapoor GS, Rohle D, Turcan S, et al. (2012) IDH mutation impairs histone demethylation and results in a block to cell differentiation. Nature 483: 474-478.
4. Prensner JR, Chinnaiyan AM (2011) Metabolism unhinged: IDH mutations in cancer. Nat Med 17: 291-293.
5. Isaacs JS, Jung YJ, Mole DR, Lee S, Torres-Cabala C, et al. (2005) HIF overexpression correlates with biallelic loss of fumarate hydratase in renal cancer: novel role of fumarate in regulation of HIF stability. Cancer Cell 8: 143-153.
6. Yang M, Soga T, Pollard PJ, Adam J (2012) The emerging role of fumarate as an oncometabolite. Front Oncol 2: 85.
7. Xiao M, Yang H, Xu W, Ma S, Lin H, et al. (2012) Inhibition of alpha-KG-dependent histone and DNA demethylases by fumarate and succinate that are accumulated in mutations of FH and SDH tumor suppressors. Genes Dev 26: 1326-1338.
8. Balss J, Meyer J, Mueller W, Korshunov A, Hartmann C, et al. (2008) Analysis of the IDH1 codon 132 mutation in brain tumors. Acta Neuropathol 116: 597-602.
9. Ross DT, Scherf U, Eisen MB, Perou CM, Rees C, et al. (2000) Systematic variation in gene expression patterns in human cancer cell lines. Nat Genet 24: 227-235.
10. illumina (2011) RNA-seq Data Comparison with Gene Expression Microarrays. illumina White Paper: illumina.
11. Schellenberger J, Que R, Fleming RM, Thiele I, Orth JD, et al. (2011) Quantitative prediction of cellular metabolism with constraint-based models: the COBRA Toolbox v2.0. Nat Protoc 6: 1290-1307.
12. Becker SA, Palsson BO (2008) Context-specific metabolic networks are consistent with experiments. PLoS Comput Biol 4: e1000082.
13. Shlomi T, Benyamini T, Gottlieb E, Sharan R, Ruppin E (2011) Genome-scale metabolic modeling elucidates the role of proliferative adaptation in causing the Warburg effect. PLoS Comput Biol 7: e1002018.
14. Folger O, Jerby L, Frezza C, Gottlieb E, Ruppin E, et al. (2011) Predicting selective drug targets in cancer through metabolic networks. Mol Syst Biol 7: 501.
15. Wang Y, Eddy JA, Price ND (2012) Reconstruction of genome-scale metabolic models for 126 human tissues using mCADRE. BMC Syst Biol 6: 153.
16. Thiele I, Swainston N, Fleming RM, Hoppe A, Sahoo S, et al. (2013) A community-driven global reconstruction of human metabolism. Nat Biotechnol 31: 419-425.
17. Wishart DS, Knox C, Guo AC, Eisner R, Young N, et al. (2009) HMDB: a knowledgebase for the human metabolome. Nucleic Acids Res 37: D603-610.
